# Advances in 3D bioprinting for regenerative medicine applications

**DOI:** 10.1093/rb/rbae033

**Published:** 2024-03-26

**Authors:** Konstantinos Loukelis, Nikos Koutsomarkos, Antonios G Mikos, Maria Chatzinikolaidou

**Affiliations:** Department of Materials Science and Technology, University of Crete, Heraklion 70013, Greece; Department of Materials Science and Technology, University of Crete, Heraklion 70013, Greece; Department of Bioengineering, Rice University, Houston, TX 77030, USA; Department of Materials Science and Technology, University of Crete, Heraklion 70013, Greece; Institute of Electronic Structure and Laser (IESL), Foundation for Research and Technology Hellas (FORTH), Heraklion 70013, Greece

**Keywords:** biofabrication, bioinks, tissue engineering, bone, cartilage, cardiovascular, neural, liver, kidney, pancreas, lungs, skin

## Abstract

Biofabrication techniques allow for the construction of biocompatible and biofunctional structures composed from biomaterials, cells and biomolecules. Bioprinting is an emerging 3D printing method which utilizes biomaterial-based mixtures with cells and other biological constituents into printable suspensions known as bioinks. Coupled with automated design protocols and based on different modes for droplet deposition, 3D bioprinters are able to fabricate hydrogel-based objects with specific architecture and geometrical properties, providing the necessary environment that promotes cell growth and directs cell differentiation towards application-related lineages. For the preparation of such bioinks, various water-soluble biomaterials have been employed, including natural and synthetic biopolymers, and inorganic materials. Bioprinted constructs are considered to be one of the most promising avenues in regenerative medicine due to their native organ biomimicry. For a successful application, the bioprinted constructs should meet particular criteria such as optimal biological response, mechanical properties similar to the target tissue, high levels of reproducibility and printing fidelity, but also increased upscaling capability. In this review, we highlight the most recent advances in bioprinting, focusing on the regeneration of various tissues including bone, cartilage, cardiovascular, neural, skin and other organs such as liver, kidney, pancreas and lungs. We discuss the rapidly developing co-culture bioprinting systems used to resemble the complexity of tissues and organs and the crosstalk between various cell populations towards regeneration. Moreover, we report on the basic physical principles governing 3D bioprinting, and the ideal bioink properties based on the biomaterials’ regenerative potential. We examine and critically discuss the present status of 3D bioprinting regarding its applicability and current limitations that need to be overcome to establish it at the forefront of artificial organ production and transplantation.

## Graphical abstract

**Figure:**
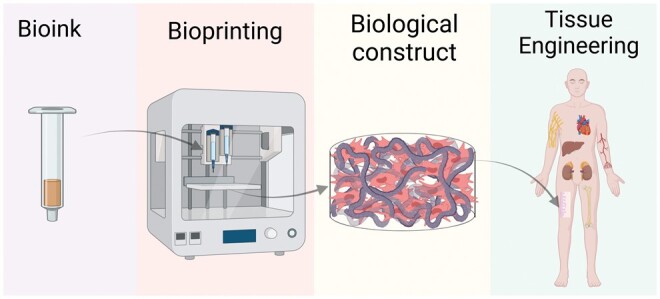

## Introduction

One of the main goals of tissue engineering is the evolvement and refinement of biofabrication techniques, in order to allow for the preparation of constructs serving as biofunctional platforms for tissue regeneration. Some of the most conventional methods for engineering such three-dimensional (3D) biocompatible environments include freeze-thawing [1], self-assembly [2], melt electrowriting [3], electrospinning [4] and freeze drying [5]. However, the aforementioned techniques cannot accommodate a spatially controlled production process, thus often being subpar in regards to reproducibility while presenting limited upscaling capability. To attenuate these shortcomings, 3D printing technologies have emerged as an alternative scaffold fabrication method, able to create scaffolds of desired geometrical traits [6–8]. 3D printers operate in tandem with computer-aided design (CAD) systems, which can provide specific spatiotemporal guidelines in the form of g-code, rendering possible the production of 3D scaffolds with highly accurate structure.

Organ models are extremely intricate biological systems that depend on the orchestrated activity of a multitude of biochemical molecules that are able to communicate and participate in various molecular pathways. Additionally, cells in organic tissue are engulfed inside complex biological macromolecular networks, making the development of *in vitro* biomimetic models even more challenging. 3D bioprinting is a relatively new biofabrication method that enables the intermixing of biocompatible materials and biological constituents as a singular entity referred to as bioink, and its subsequent 3D printing into structures of tailored architecture, following automated protocols [9–11]. Although bioprinting is still in its early stages of development, it has amassed great amounts of interest inside the research community, as it can be employed to construct an artificial environment that closely resembles that of the native tissue. One of the main advantages of this technique is that it not only allows the fabrication of constructs with desirable mechanical properties, comparable to the tissue type that we aim to restore, through layer-by-layer deposition of the respected bioink solution into a hierarchical pattern, but also controls the cellular density of the produced construct, which is a crucial parameter to mimic for any potential medical device [12, 13]. Moreover, it has enabled the prospect of augmented *in situ* therapy, with the use of fully automated robotic technologies, conferring maximum precision while simultaneously minimizing the involvement of human element in the process [14]. To develop a model that is eligible to replicate the high cellular density and bioactivity of scarred myocardial tissue after myocardial infarction, Daly *et al.* [15] prepared microspheres comprising high concentrations of induced pluripotent stem cell (iPSC)-derived cardiomyocytes and primary human cardiac fibroblasts (CF). With the use of a support hyaluronic acid (HA)-based hydrogel, microspheres of the two cell populations were 3D bioprinted onto the scaffolds’ surface in a circular pattern. The high cellular density of the adjacent microspheres allowed for their spontaneous merging into soft biofunctional microtissue tubes, resulting in the fabrication of a potent co-culture system for tissue regeneration and drug screening applications.

So far, bioink hydrogels have been developed by combining different natural biomaterials such as gelatin [16], alginate [17], fibrin [18], HA [9] and extracellular matrix (ECM) [19], as well as a variety of synthetic biopolymers including polyvinyl alcohol (PVA) [20], polyvinyl pyrrolidone (PVP) [21] and polyethylene glycol (PEG) [22]. Additionally, the incorporation of biofunctional nanoparticles into bioprinting suspensions is usually favored for the construction of 3D bioprinted constructs, as, even in small quantities, they can drastically alter the biological and mechanical response of the resulting bioinks [23, 24]. However, since the number of available biomaterials for bioprinting is finite, there is an ever-increasing need for new material combinations, as well as innovative approaches focusing on the pre and post-processing stabilization of the 3D bioprinted constructs [25]. As most bioink solutions comprise materials that can absorb large amounts of water, therefore resembling hydrogel solutions, they often suffer from poor printing fidelity and accuracy, which stem from the immiscibility of the base constituents and the high surface tension forces that are built, leading to premature scaffold contraction and, ultimately, to collapse [26, 27]. To improve printability, different methods have been suggested, such as the utilization of bioinks with self-assembly properties [28] or the addition of biopolymers to bioinks for enhanced rheological properties [29].

This review aims to underline the recent advances in the area of 3D organ bioprinting, based on the biomaterials’ chemical composition and bioprinting techniques. Our main goal focuses on how various bioprinting methodologies have been employed to produce 3D constructs for the regeneration of different tissue types, including bone, cartilage, neural, cardiovascular, skin and organs such as liver, kidney, pancreas and lungs, using mono and co-cultures, while also presenting a critical perspective on their scientific impact. Moreover, the ideal bioink properties and the basic types of 3D bioprinting are reviewed. In the last section, we critically discuss the hurdles that contemporary 3D bioprinting encounters, and current technological limitations that need to be overcome, so that bioprinting expands its full potential towards the ultimate goal of producing functional artificial organs, while also providing insight on how this could be attained.

### Bioink requirements

The gelation, rheological, mechanical and biological properties of a bioprinting solution are all equally important, as each parameter plays a critical role in ensuring the successful printability, mechanical strength and biofunctionality of the resulting scaffolds [30]. In particular, gelation at room temperature is a vital characteristic for any potential tissue engineering platform, as it is necessary for the maintenance of the final structure and the prevention of deformation of the 3D printed hierarchical status in an *in vivo* environment. It can manifest either spontaneously, as a result of the innate gelation capacity of the bioink components, or by introducing exogenous agents in the solution which can induce chemical cross-linking [31]. However, the gelation process should be controllable in order to avoid problems during the printing procedure [32].

Rheological properties, such as viscosity and shear thinning behavior, are inextricably tied to the printability of a particular solution. A bioink should have enough viscosity to enable proper flow with minimal obstructions, as well as enough cohesion so that it does not bend or break down into smaller segments during the printing process [33]. The shear thinning effect appears in non-Newtonian fluids and is expressed as the viscosity’s tendency to decline when external shear stresses are applied to them. Shear thinning curves can prove extremely useful in 3D bioprinting techniques to compare the bioinks’ shear rates at the same viscosity levels. This comparison may lead to a decision, on which bioink would be extrudable at lower pressures, ensuring higher cell survival. A thixotropic behavior can also allow bioinks to more easily retain their shape, as they become more resistant to deformation from mechanical stresses, giving them the necessary adaptability for medical applications [34]. The main tailorable parameters for the control of rheological characteristics include the nature of the bioink components, their respective concentrations and molecular weights, as well as other conditions under which the 3D bioprinting is conducted, such as the temperature and application of external mechanical stimuli [35, 36].

As mentioned, a bioink ought to depict enough robustness in order to mimic the mechanical characteristics of organic tissue and to be able to provide the necessary support for cell growth and survival [37]. However, there is an inverse relationship between a 3D bioprinted scaffold’s mechanical strength and its biological response, as the stiffer the material, the less free space cavities are available for the engulfed cells’ infiltration and thus expression of their biofunctionality. Additionally, a dense material inner core may impose restrictions on cells’ signals exchanging and evoke issues of limited diffusion of nutrients, reducing the cytocompatibility status and the overall regenerative efficacy of the implant [38, 39]. Biodegradability is another crucial factor to take into consideration when designing a 3D bioprinted construct composition, as the material should degrade gradually, allowing the cells to formulate their innate ECM before its total dissolvement [40]. It is also important to underline that, although some biomaterials can present cytocompatible character, they may not be able to degrade under *in vivo* conditions, thus requiring further modifications before they can be reconsidered for bioprinting applications [41].

Biocompatibility and biofunctionality are the most essential components for a bioink, as they constitute the main determinants of its ability to initiate, progress and enhance the *de novo* tissue regeneration process. The term biofunctionality refers to the capacity of a bioink to direct or amplify a wide range of cellular biological modalities, extending from the differentiation guidance towards desired cell lineages to the activation of particular molecular pathways [42]. For this reason, biomolecules with distinct functionalities such as growth factors or cytokines, which are able to interact with the cells in a timely controlled manner and promote particular cell behavior vital for tissue reconstitution [43]. There is also a large variety of biomaterials which are widely used in bioprinting as they not only depict cytocompatibility but can also act as boosting mediators of cellular biofunctional properties, while concurrently being able to positively affect the scaffold’s structural integrity [23]. Table 1 shows representative biomaterials used as bioinks for the regeneration of specific tissue types focusing on the *in vitro* results.

**Table 1.** Biomaterials utilized as bioinks for various tissue engineering applications focusing on *in vitro* results

Biomaterials	Technique	Tissue	Cell type	Viability	*In*/*ex vivo* model	Ref
Alg + Fib	Extrusion	Neural	hMSCs	>90% at 9 days	—	[44]
Alg + GO	Extrusion	Bone	hMSCs	>75% at 7 days	—	[45]
Alg + Gel	Extrusion	Neural	NKCs	∼80% at 4 days	—	[46]
Alg + Gel + GO	Extrusion	Bone	hMSCs	>85% at 42 days	—	[47]
ADA + Gel	Extrusion	Endothelial/neural	HUVECs/RSC96	>70% at 7 days	—	[48]
PVA + dECM	Extrusion	Cartilage	iPSCs	>75% at 7 days	—	[20]
PEG + HA	Extrusion	Cartilage	hMSCs	98% at 0 day	—	[49]
PEGDA	Inkjet	Cartilage	hMSCs	63% at 0 day	—	[22]
PEGDA + Laponite	Extrusion	Bone	ROBs	>95% at 0 day	Rat	[50]
Gel + Alg	Extrusion	Neural	RSC96	>90% at 7 days	Mouse	[51]
GelMA	Lithography	Cornea	hCSs	82% at 1 day	—	[52]
GelMA + Alg + AlgS	Extrusion	Cartilage	pMSCs	∼70% at 42 days	Mouse	[53]
GelMA + Alg + TCP	Extrusion	Osteochondral	hMSCs	>70% at 21 days	—	[54]
GelMA + fibrin	Extrusion	Liver (vascularized)	HUVECs + hMSCs	80% at 7 days	Rat	[55]
GelMA + laponite	Extrusion	Bone	hBMSCs	86% at 21 days	CAM	[56]
GelMA + PEGDA	Lithography	Cartilage	hMSCs	75% at 1 day	—	[57]
GelMA + PEGDA + PEDOT: CSMA + TA	Extrusion	Neural	rNSCs	>85% at 0 day	—	[58]
GelMA + Gel + HAP	Extrusion	Bone	MC3T3-E1	>76% at 1 day	—	[59]
HAMA	Lithography	Endothelial	HUVECs	90% at 0 day	—	[60]
PU	Extrusion	Neural	hDFs	>90% at 14 days	—	[61]
SF + dECM	Extrusion	Cartilage	rMSCs	98% at 0 day	Mouse	[62]
dECM	Extrusion	Heart	NRCM	>97% at 14 days	—	[63]
dECM + PEG	Extrusion	Liver	HepG2 + NIH 3T3	>90% at 7 days	—	[64]
dECM + Laponite + PEGDA	Extrusion	Cardiac	hCFs	>90% at 7 days	—	[65]

dECM, decellularized extracellular matrix; alg, alginate; AlgS, alginate sulfate; ADA, alginate dialdehyde; fib, fibrinogen; gel, gelatin; GelMA, gelatin methacrylate; GO, graphene oxide; HAP, hydroxyapatite; Hama, hyaluronic acid methacrylate; PVA, poly(vinyl alcohol); PEDOT, poly(3,4-ethylenedioxythiophene); CSMA, chondroitin sulfate methacrylate; PEGDA, poly(ethylene glycol) diacrylate; PU, polyurethane; TA, tannic acid; TCP, tricalcium phosphate; hBMSCs, human bone marrow stromal cells; MC3T3-E1, mouse calvarial pre-osteoblastic cells; hCSs, human corneal stromal cells; hMSCs, human mesenchymal stem cells; pMSCs, porcine mesenchymal stem cells; rMSCs, rabbit mesenchymal stem cells; rNSCs, rat neural stem cells; hDFs, human dermal fibroblasts; CAM, chorioallantoic membrane; ROBs, primary rat osteoblasts; HUVECs, human umbilical cord endothelial cells; hCFs, human cardiac fibroblasts; NRCM, neonatal rat cardiomyocytes; RSC96, rat Schwann cells; HepG2, human liver cancer cells; NIH 3T3, mouse fibroblast cells; NKCs, natural killer cells.

### 3D Bioprinting techniques

#### Extrusion-based 3D bioprinting

Extrusion-based 3D bioprinting (EBB) constitutes the most common bioprinting modality, utilizing either pneumatic or mechanical-driven dispensing systems. In a pneumatic-driven system, pressurized air provides the required force that is necessary to extrude a bioink composition while mechanical-driven systems employ pistons or screw plungers to generate extrusion forces [66]. EBB strategies are also able to incorporate multiple printing heads to allow for concurrent 3D bioprinting of distinct solutions forming one singular complex 3D construct [67]. EBB’s high popularity and extensive usage are due to its feasibility to print hydrogels of a wide range of viscosities (30–6 × 10^7^ mPa s) and fabricate large-scale models (centimeter magnitude) with high cell densities (10^8^ cells/ml) [68]. Although this technique is mostly praised due to the high extrusion pressures, it can attain, it suffers from limited printing accuracy, while shear stresses induced by the nozzle geometry and high applied pressures that correlate to the viscosity of the bioink can significantly influence cell viability during the printing phase [69, 70]. Οn this subject, Ning *et al.* [71] reported ∼95% viability with 50 kPa pressure on both Schwann cell line (RSC96) and myoblast cell line (L8) post-printing, which dropped down to ∼80% at 400 kPa whereas Chung *et al.* [72] isolated primary myoblasts which maintained over 90% viability, 1 h after printing and didn’t find any significant differences among 55 and 165 kPa pressures.

#### Jetting-based bioprinting

In jetting-based bioprinting, the bioink is stored in a liquid phase inside a specially designed chamber and it is then deposited in a drop-wise manner towards a surface area following a particular 3D architecture. The jetting technologies differ based on the physical principles that are responsible for the generation of the droplet. The most common jetting mechanisms rely on thermal, piezoelectric and light-based sources [73]. In thermal jetting, the heat actuator surrounds the fluid chamber and generates a heat bubble which in turn propels the fluid through the nozzle in the form of small droplets. In piezoelectric systems, a piezoelectric ceramic material coating surrounds the chamber and when it receives the necessary electrical pulse, a sudden deformation of the chamber wall occurs which leads to the alteration of the fluid volume, creating the final bioink droplet formation [74]. Moreover, in light-based techniques a laser beam is focused on a donor-slide and, by pulsing, it generates a high-pressure bubble which propels bioink droplets onto a receiver-slide [75]. Overall, jetting-based bioprinting requires low viscosity solutions in order to work but facilitates good printing accuracy and enhanced printing speed, while maintaining high levels of biocompatibility (>80%) although challenges regarding the viability of the cells still persist as the polymer concentration, the impact velocity and the size of the droplet are all contributing factors [76, 77].

#### Vat polymerization-based bioprinting

In vat polymerization bioprinting, a light source selectively polymerizes or crosslinks a monomer- or prepolymer-based bioink (bioresin) that rests on a digitally controlled platform. Depending on the way light is guided, there are several bioprinting technologies. Typical stereolithography setup includes a light source, optics, a scanning galvanometer, a bioresin reservoir and a platform where polymerization is achieved linearly, while in digital light processing (DLP), a digital mirror replaces the scanning galvanometer, and the photocuring occurs planarly [78, 79]. In order to build up 3D dimensional structures, these techniques are based on the constant alteration between two phases, the bioink printing phase inside the reservoir mold and the photocuring phase, to solidify each subsequent layer. This in turn leads to slower printing times but higher printing resolutions [52]. On the other hand, two-photon polymerization utilizes a near-infrared femtosecond laser which emits a tightly focused beam onto the bioresin reservoir. By moving the focal point of the laser, a 3D structure can be fabricated with a resolution beyond the optical diffraction limit. The selection of the appropriate biocompatible photoinitiators will determine the wavelength of the laser which in turn can affect cell viability as UV light may harm cells [80, 81]. Table 2 summarizes the characteristics and comparison of 3D bioprinting technologies.

**Table 2.** Characteristics and comparison of 3D bioprinting technologies

	Cell densities	Speed	Resolution	Viscosity	Quality of vertical structure	Cost	Commercial availability	Ref
Extrusion	10^8^–10^9^ cells/ml	10–50 μm/s	100 μm	30–6 × 10^7^ mPa/s	High	Low	Wide	[82–85]
Jetting-based	10^6–^10^8^ cells/ml	Up to 10^4^ droplets per second	10–50 μm	3–300 mPa/s	Limited	Low	Wide	[73, 84–86]
Vat polymerization	<10^7^ cells/ml	700 mm/h	10 μm	<5000 mPa/s	Moderate	Moderate	Limited	[79, 84, 85, 87]

## Applications of bioprinting in tissue and organ regeneration

### Bone 3D bioprinting

Bones are complex organs consisting of several distinct tissue types, including cortical and cancellous bone, bone marrow and periosteum. Although bone is the hardest organ in the human body, its homeostasis relies heavily on a highly dynamic and adaptive system that is prone to respond to biomechanical ques for its growth and long-term sustainability [88]. Specifically, bone tissue is composed of three specialized cell categories known as osteoblasts, osteoclasts and osteocytes, as well as a supportive ECM network comprising different types of collagenous fibers and mineral crystals, primarily based on calcium ions and phosphate groups [89]. Osteoblasts are responsible for bone formation while osteoclasts are involved in the antagonistic activity of bone resorption [90, 91]. Conversely, osteocytes, which are the most abundant of the three and the result of osteoblasts’ differentiation, play a critical role in maintaining the structural and mechanical integrity of bone tissue, by regulating bone remodeling and mineralization, and are associated with both bone formation and resorption processes [92].

The human body is physically supported by the musculoskeletal system, with bone, as the most inelastic type of tissue, possessing limited intrinsic regeneration capacity level [93]. Bone structural damage that exceeds a critical size threshold, usually requires medical intervention to restore, often followed by long rehabilitation periods. The golden standard for bone defects consists of the use of autologous bone grafts, but many complications are still prone to occur during and after the surgical procedure at the harvested tissue site, such as donor site injury, deformity, scarring and even morbidity [94, 95]. Allograft transplantation is another alternative method of treatment, with similar downsides and also increased risk of transplant rejection due to immunogenic incompatibilities [96]. Additionally, some of the more conventional strategies for bone reconstruction include the utilization of biologically inert metallic devices which have comparable mechanical properties to that of the native bone, but often require subsequent revisional surgery cycles due to the material’s susceptibility to wear and corrosion [89, 97].

3D bioprinting offers the possibility to create patient-specific implants that match the geometry and mechanical properties of the respective fractured bone area [98], by being able to create multi-layered structures that mimic the complex architecture of natural bone tissue, which can improve the stability of the implant [99, 100]. This in turn can lead to increased chances of transplant assimilation by the surrounding tissue, reduce rejection rates and hasten the total healing process [101]. Li *et al.* [102] demonstrated the ability to repair large segmental bone defects in swine, by *in situ* extrusion bioprinting. To prepare the bioink, pre-crosslinked sodium alginate was mixed with a solution of polyethylene glycol diacrylate (PEGDA), gelatin methacryloyl (GelMA) and MC3T3-E1 pre-osteoblastic cells, and was then photopolymerized through UV curing. The scaffolds showcased high mechanical stability, as well as great printing accuracy. To evaluate the bioink osteogenic potential, ALP, COL1, OCN and RUNX2 gene expression was investigated. The PCR analysis illustrated an upregulation of all markers at Days 7 and 21, compared to control cell culture plates. After 12 weeks of implantation, the bone over tissue volume ratio was significantly elevated in the bioprinted group, with the micro-computed tomography (micro-CT) scans depicting the development of smooth bone surface. Additionally, Goldner’s trichrome staining revealed that in the animals that were treated with the bioprinted construct, the while a disordered morphology and small defects were formed in the control group, in the bioprinted group the morphology was improved and osteoblasts were arranged homogenously and compactly throughout the defect region.

**Figure 1.**
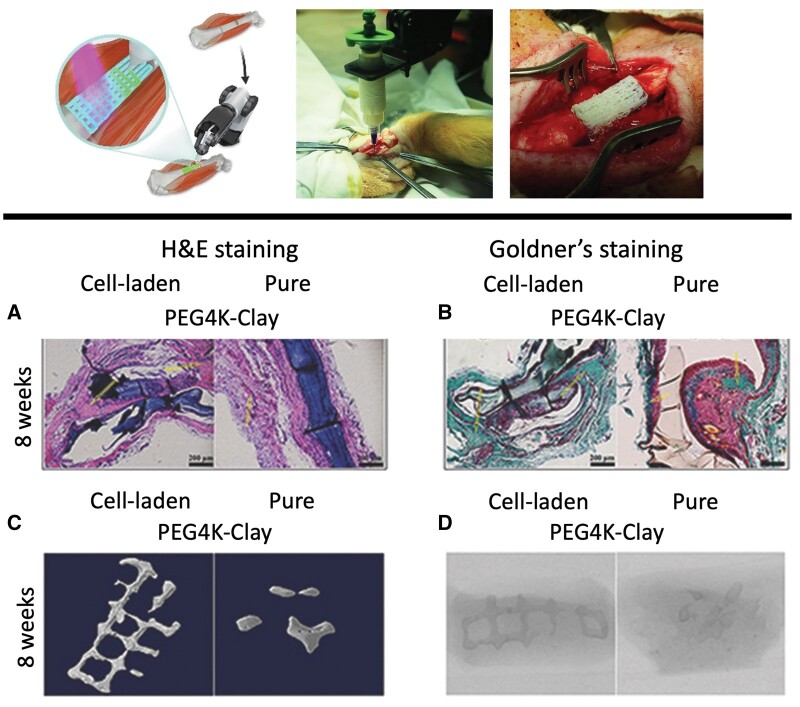
*In situ* extrusion bioprinting process in pig model with generated defects on the right tibia (upper panel). The procedure of *in situ* 3D bioprinting in *in vivo* model with the final product (scaffold with porous structure) is illustrated from left to right (reproduced with permission from [102]). Primary rat osteoblasts in PEGDA–laponite–hydroxyapatite composite construct in ectopic osteoinduction experiments in rats (lower panel). Histological staining observations after 8 weeks of implantation. H&E staining (**A**) and Goldner’s staining (**B**) visualize new bone tissue in cell-laden PEG4K–clay and pure PEG4K–clay groups. The formation of new bone was verified using reconstructed 3D models (**C**) and micro-CT images (**D**) obtained 8 weeks post-surgery. Scale bars represent 200 μm (reproduced with permission from [50]).

As the environment of natural bone contains minerals such as calcium, magnesium and phosphorus, bone cells’ functionality strictly depends on the uptake and utilization of these compounds, with their presence having a regulating effect on their proliferation and differentiation capacity [103, 104]. Therefore, their incorporation inside bioinks to prepare osteogenesis promoting scaffolds has attracted significant attention. In particular, hydroxyapatite (HAP) is the chief mineral of native bone, which is rich in calcium and phosphoric groups, and has been extensively used in bone tissue engineering due to its osteoinductive capability and its mechanical strength [105, 106]. In this context, Keriquel *et al.* [107] evaluated the impact of printing geometry in the distribution of viral transduced multipotent mouse bone marrow stromal precursor D1 cells and performed *in vivo* and *in situ* bioprinting on mice. Using a LAB setup, cells were mixed with collagen and nano-hydroxyapatite (nHAP) to form the bioink which was subsequently 3D bioprinted following two distinct architectural patterning designs, a 2-mm diameter disk formation and a ring with an outer and inner diameter of 3.0 and 2.1 mm, respectively. Both structures showed similar cell proliferation profile, while the micro-CT and histological analysis of the samples revealed that the nHAP-collagen bioinks of disk shape, formed extensive and homogenous bone tissue throughout the defect area after 2 months, whereas those printed in ring geometry favored the bone reconstitution process mainly in the perimeter. In another bioprinting study, using an extrusion-based bioprinter, Allen *et al.* [59] studied the effects of four different concentrations of HAP (0, 5, 10 and 20 mg/ml) on the growth and differentiation of MC3T3-E1 pre-osteoblastic cells when incorporated in GelMA-gelatin 3D bioprinted constructs. All concentrations up to 20 mg/ml of HAP proved to be fully cytocompatible, with negligible differences among them. Alkaline phosphatase activity (ALP) assessment was used as a marker for the investigation of osteogenic differentiation. The addition of 5 and 20 mg/ml of HAP significantly increased ALP expression at Days 7 and 28. Cell viability was measured by using live/dead staining with calcein-AM and ethidium homodimer-1 at Day 1, which showed that only a very small portion of the initial cell number was lost during the bioprinting and the photo-crosslinking processes. Interestingly enough, confocal microscopy images revealed a pattern of increased cell concentration in the periphery of the hydrogels compared to the more central areas, indicating that GelMA may not having actually facilitated significant cellular migration. Osteogenic gene expression was quantified using RT-qPCR. It was found that the constructs containing 20 mg/ml HAP presented the highest expression out of all compositions and significantly higher upregulation of both BMP7 and osteocalcin osteogenic markers at Days 14 and 28, compared to the control non containing HAP bioinks, suggesting this particular concentration to retain the optimal osteogenic response. Laponite is another very popular biocompatible bioceramic that consists of bioactive silicate nanoplatelets that contain, among others, magnesium and sodium ions, whose presence has been correlated to the boosting of osteogenesis process [56, 108]. Zhai *et al.* [50] developed two bioinks comprising 7% w/v laponite nanoparticles, 20% w/v HA and 20% w/v PEGDA of two distinct molecular weights, 4K (PEG4K) and 10K (PEG10K), mixed with primary rat osteoblasts (ROBs). The bioprinted constructs were fabricated utilizing an extrusion bioprinter and crosslinked via UV irradiation for 10 min. The viability of the encapsulated cells was measured after 7 days of culturing, by staining them with calcein-AM and EthD-1. As control, the same composition scaffolds were used which were cell-seeded instead of being 3D bioprinted. The bioinks presented significantly higher cytocompatibility profile than the cell-seeded scaffolds, at all time points and for both molecular weights of PEGDA. However, between the two PEGDA molecular weights, the PEG4K retained the highest viability level, probably due to its significantly less stiff nature compared to the PEG10K ones. ALP activity assay in the bioprinted constructs showed that the ROBs within the PEG4K-clay composites had significantly elevated enzymatic activity at Days 7, 14 and 21 of culture, compared to their no laponite containing counterparts, with the ALP staining findings at Day 21 validating these results. To further evaluate the osteogenic capabilities of the scaffolds, the PEG4K-clay bioink was used for *in vivo* tibia repair and ectopic osteoinduction experiments on SD mice. Through sequential fluorescent labelling, the formation of new bone was observed after 8 weeks and it was found to be more abundant and more continuous in the cell-laden PEG4K-clay composites compared to the pure PEG4K-clay scaffolds and the control group. Furthermore, micro-CT images showed that most of the newly constructed bone tissue could be located primarily in the marrow cavity, around or inside the ROB-laden PEG4K-clay constructs. The generation of new osteoblasts and fibroblasts was identified by Giemsa/H&E staining. For the monitoring of the cell viability in the ectopic experiments, ROBs were conjugated with green fluorescent protein. After 21 days of implantation, no obvious immunoreaction occurrence was evident, as suggested by the large number of alive cells. H&E and Goldner’s staining were also used to evaluate the osteoinductive properties of the constructs. After 8 weeks of implantation, it was revealed that the PEG4K-clay nanocomposite bioinks retained the largest and thickest new bone tissue formulation, out of all examined conditions. Figure 1 presents two examples of bioprinting for bone tissue regeneration using extrusion bioprinting in a pig model with generated tibia defects and PEGDA–laponite–HAP with ROBs constructs in an ectopic osteoinduction experiment in rat.

Graphene oxide (GO) is a two-dimensional material consisting of a single layer of carbon atoms arranged in a hexagonal lattice format [109]. It demonstrates a wide array of useful properties, such as high surface area, competent mechanical strength, electrical conductivity, antibacterial and biocompatible properties, while also depicting satisfactory levels of dispersion in water-based environments, making it an excellent option for bioprinting applications [110–112]. Choe *et al.* [45] studied the effect of GO’s presence on human mesenchymal stem cells (hMSCs), in regards to their proliferation and osteogenic induction potential, by preparing a series of bioinks after mixing GO in different concentrations, up to 1 mg/ml, with a standard concentration of 3% w/v alginate. The scaffolds were pre-crosslinked after immersion in a CaCl_2_ solution, to achieve sufficient printability. Immediately after bioprinting, the cell-laden constructs were crosslinked furtherly with 90 mM of CaCl_2_ for 3 min, washed and eventually placed in culture medium. Cell viability examination was performed over a period of 7 days, by staining the constructs with calcein-AM and ethidium homodimer. Furthermore, to study the survival of the cells under oxidative stress, cell medium was supplemented with 400 mM of H_2_O_2_. No significant differences in cell viability were observed among the different concentrations of GO, whereas under oxidative stress conditions, the GO-containing bioinks proved to adapt better, by retaining higher cytocompatibility levels compared to control alginate bioinks, due to GO’s antioxidant activity. PCR analysis of ALP, BSP, OPN and Runx2 markers showcased their upregulation in all GO-containing bioinks at Day 7. However, after 14 days, the 1 mg/ml GO bioinks displayed a significant decrease in ALP expression, while the control and 0.5 mg/ml GO compositions depicted an elevated expression of the BSP gene. Moreover, there were no significant differences in the expression of OPN gene between the GO-containing constructs, as they retained comparable levels to those attained till Day 7. The differentiation efficacy of hMSCs was also investigated through Alizarin Red staining in the biofabricated constructs after 7 and 14 days of culture. At Day 7, the GO-free constructs showed low degrees of mineralization, with the GO counterparts depicting similar levels between them but higher compared to control. In particular, the 0.25, 0.5 and the 1 mg/ml GO constructs were the most vividly stained out of all compositions. As mentioned, GO possesses sufficient mechanical strength, even at lower concentrations, allowing it to be used as modulator for the robustness of the scaffolds it is incorporated into. Based on this attribute, Zhang *et al.* [47] investigated how the printability of a alginate/gelatin based-bioink containing hMSCs could be affected by the addition of various concentrations of GO, up to 2 mg/ml. The cell-laden constructs were bioprinted using an extrusion bioprinter and crosslinked with a solution of 2% w/v CaCl_2_ for 10 min. Confocal staining with calcein-AM and ethidium homodimer presented comparable levels of biocompatibility among the different bioinks, up to Day 42, without significant cell number decrease. ALP activity analysis revealed that the 1 and 2 mg/ml GO groups had a significantly higher number of *p*-nitrophenol units over DNA content, compared to the control and 0.5 mg/ml GO groups, suggesting the GO’s role in osteogenesis. PCR for bone-related genes was also conducted after 42 days. Specifically, ALPL and BGLAP expression was significantly upregulated in the 0.5 and the 1 mg/ml GO containing bioinks, compared to control, while PHEX gene expression was significantly increased in the 1 mg/ml GO scaffolds, compared to the 0.5 mg/ml GO and control groups. Using micro-CT imaging technique, the mineralization volume was monitored after 42 days of culture. The 1 mg/ml GO constructs demonstrated the highest mineral volume at Days 35 and 42, while having significantly higher mineral volume than the control scaffolds, at all examined time points. Hematoxylin and eosin (H&E) staining showed that cells had spread uniformly in all groups but it is noteworthy to note that, in the control scaffolds, they had adopted a smaller and more elongated shape, whilst those engulfed in the GO containing bioinks were larger and of polygonal shape, resembling more the characteristic appearance of mature osteoblasts.

### Cartilage 3D bioprinting

Cartilage is a flexible connective tissue that provides an elastic and low friction interface area between bone and joints. As such, its main goal is to evenly distribute the various mechanical stresses that the native bone tissue is subjected to on an everyday basis, as bone’s intrinsic sturdiness allows for limited flexibility and, without cartilage’s contribution in the attenuation of these external compressive forces, would be much more prone to fractures and various traumas [113, 114]. Based on chemical composition and topography, cartilage can be divided into articular cartilage, fibrocartilage and elastic cartilage [115]. Between the three types, articular cartilage, which is located at the end surfaces of native bone, is the most susceptible to injury damage [116–118]. It chiefly consists of three distinct zones: (i) a thin superficial layer designated as tangential, mainly made of collagen type II and IX, that constitutes up to 10–20% of the total thickness of cartilage tissue, which can act as the first barrier against different mechanical loads and provide sufficient mechanical support to the inner articular cartilage core, (ii) a middle layer designated as transitional, which makes up to 40–60% of articular cartilage thickness and comprises a large variety of collagen and proteoglycan mixtures, displaying a tight and packed spatial organization in order to increase cartilage’s mechanical strength and (iii) an inner layer designated as deep zone, which has the highest concentration of collagen and proteoglycan fibers and the lowest water content, out of the three, taking up to 30% of the total articular cartilage volume and possessing the highest mechanical resistance among the three layers [114, 119, 120].

Despite its involvement in many complex biomechanical systems, cartilage lacks an autonomous blood and lymph supply network, thus it has to rely on a local diffusion from the adjacent environment model for its nutrition and its long-term sustainability. Another important parameter that significantly impairs cartilage reconstitution capacity is the low cellular density it possesses, making the self-healing process even more difficult [114]. Cartilage pathophysiology has also been found to present strong correlation with osteoarthritis, one of the most prevalent and thoroughly studied disease models [121–123]. Due to all these particularities, cartilage regeneration techniques have rapidly evolved, especially those revolving around the restoration of articular cartilage, ranging from the use of novel methods based on pluripotent stem cell therapy [124–126], to the utilization of chondrogenesis related growth factors such as fibroblast growth factor [10], transforming growth factor-β (TGF-β3) [127] and insulin-like growth factor-1 (IGF-1) [128], as well as the employment of biomaterial-based scaffolds [129].

3D bioprinting technology presents a very promising avenue for the restoration of cartilage tissue, by trying to fabricate biomimicking constructs that meet the requirements that stem from the metabolic and structural distinctiveness of each cartilage type tissue [9, 11, 130]. As in 3D printing, bioceramics are considered to be one of the most popular categories of biomaterials for cartilage 3D bioprinting applications, providing the possibility to not only fine-tune the biological but also the mechanical properties of the resulting bioink [131, 132]. Another study employed the use of β-tricalcium phosphate (TCP) microparticles to design 3D bioprinted constructs for the restoration of calcified articular cartilage tissue [54]. To evaluate the role of the microparticles in chondrogenesis, the team prepared two types of bioinks embedded with bone marrow-derived human mesenchymal stem cells (BMSCs), with the first one consisting of GelMA and alginate and the second one with the exactly same polymeric composition but with the additional implementation of β-TCP microparticles. The chondrogenic capabilities of the bioinks were assessed over a period of 3 weeks of cell culture. The scaffolds were first physically crosslinked by submerging them in a CaCl_2_ solution in order for the gelation of alginate to take place and then by photopolymerizing the methacrylated groups of GelMA with the use of Irgacure 2959 photoinitiator. The rheological analysis revealed that both bioinks possess shear-thinning behavior, with the presence of β-TCP microparticles leading to enhanced printability, as well as significantly higher viscosity which peaked at 300 Pa s, while the control scaffolds maximum value was 3.2 Pa s. The BMSCs viability was assessed after 3 h, 7 and 21 days. Bioinks depicted similar biocompatibility at all investigated time points, with only a marginal decline between 3 h and day 21, indicating their suitability as a potential environment for long term cell growth. Additionally, a series of cartilage specific markers were examined by means of confocal microscopy and PCR, among them being collagen Type 2 (COL-II), collagen Type 10 (COL-X) and aggrecan (ACAN). Microscopy images showed that the abovementioned markers were expressed in both bioink types but PCR analysis illustrated that the β-TCP containing bioink had significantly higher gene upregulation, for each marker.

In order for the cells to be able to grow in number and proliferate inside the artificial matrix of a 3D bioprinted scaffold, a highly porous interior environment is required, with the formation of cavities and interconnected corridors that resemble the structure of the native cartilage tissue. On top of that, as cartilage is constituted by different layers, the parameter of zone-specific chondrogenesis through the prism of 3D bioprinting has amassed great interest in the recent years [133]. In one such work, Mouser *et al.* [134] tried to focus on the fabrication of bioinks that can separately promote the regeneration of the superficial, middle and deep zone of the articular cartilage. To achieve this, they used three distinct biomaterial compositions, GelMA/gellan gum (GG), GelMA/gellan gum/methacrylated hyaluronic acid (GGH) and GelMA (G) acting as control. Additionally, three types of cells, articular cartilage progenitor cells (ACPCs), multipotent mesenchymal stromal cells (MSCs) and mature chondrocytes, were used for the production of the final bioinks after photopolymerization, to deduce which cell category has the optimal regenerative capacity for each zone. The quantitative analysis for the glycosaminoglycan (GAG) content showed that GAGs formation was similar between the GG and GGH but substantially higher in the case of ACPCs and MSCs-laden bioinks when compared to the chondrocytes-laden ones. Moreover, the qualitative histological analysis for the identification of GAGs and COL2 formation, also validated the increased effect of the MSCs and ACPCs compared to mature chondrocytes. ACAN staining at Day 28 revealed that the GG MSCs-laden scaffolds had the densest aggrecan network formulation, while the COL2 staining images also suggested the same. For the evaluation of specific zone effect, the GGH hydrogel with ACPCs was selected for the 3D bioprinting of the superficial region and the GGH hydrogel with MSCs was selected for the fabrication of the middle/deep region. Apart from the singular zone bioprinted constructs, two-zone constructs were also bioprinted so that their biological response could be correlated to that of the singular ones, designated as two-zone superficial and two-zone middle/deep, respectively. The biological impact of each of the four scaffolds was examined by PCR. ACAN gene at Day 28 was most upregulated by two-zone middle/deep bioinks while at Day 42 by the two-zone superficial. The exact same motif of gene expression could be detected for COL2 marker. Moreover, at Day 42, the two-zone superficial composition clearly exceeded the rest in regards to the expression of proteoglycan Type 4, while COL10 marker had the highest expression levels in the case of two-zone superficial scaffolds, at Day 28. All these findings suggest the superior biological effect of the two-zone constructs compared to the singular ones, underlying the importance of zone-specific 3D bioprinting and how its advancement can lead to the optimization of articular cartilage reconstitution.

The transforming grow factor superfamily encompasses a wide group of divergent biomolecules, able to act as regulating agents for a plethora of biological processes such as cellular proliferation, cell lineage directionality, formation of ECM and communication between different cell types [135, 136]. In regards to cartilage tissue formulation, it has been shown that the subfamily of TGF-beta variances can significantly affect pluripotent cells’ differentiation towards mature chondrocytes [127, 137]. As such, the encapsulation of TGF-beta molecules as chondrogenesis boosting factors inside a 3D bioprintable construct has been a commonly utilized strategy in the last years, with the most thoroughly investigated types being TGF-beta1 and TGF-beta3 [49, 57, 138]. Based on that philosophy, Wang *et al.* [53] prepared a GelMA/sulfated alginate/TGF-beta3 bioink infused with BMSCs, in order to increase the binding affinity of TGF-beta3 through the presence of sulfated groups, thus better control its release rate and, evidently, the chondrogenic response. Additionally, a GelMA/alginate/TGF-beta3 composition was also 3D bioprinted to act as control. The two scaffold types did not depict any difference in their viscosity, shear-thinning and thixotropic behavior, retaining similar printability and shape fidelity. The BMSCs biocompatibility revealed similar patterns for the two bioinks, but the TGF-b3 release profile, which was monitored via ELISA assay, showed a steady release of TGF-b3 from the GelMA/sulfated alginate/TGF-b3, with the cumulative release being below 40% of the total growth factor content by Day 7, while the GelMA/alginate/TGF-b3 scaffolds exhibited an initial burst release of TGF-b3, which reached up to 60% by Day 3. To evaluate the role of TGF-b3 encapsulation in chondrogenesis compared to being externally supplied, two more non-containing TGF-b3 bioinks were prepared, with the same polymeric concentrations, which were cultured in TGF-b3 containing cell medium. The *in vitro* staining evaluation showed that the GelMA/sulfated alginate/TGF-b3 bioinks had higher GAGs content and the GelMA/alginate/TGF-b3 counterparts higher calcium content, when compared to their externally supplemented TGF-b3 counterparts, respectively. The GelMA/sulfated alginate/TGF-b3 and GelMA/alginate/TGF-b3 bioinks were subcutaneously implanted in mice over a 4-week period. The immunohistochemical and histological analysis of sGAG, collagen, calcium, collagen type II and collagen type X validated the optimal effect of the GelMA/sulfated alginate/TGF-b3 bioinks.

**Figure 2.**
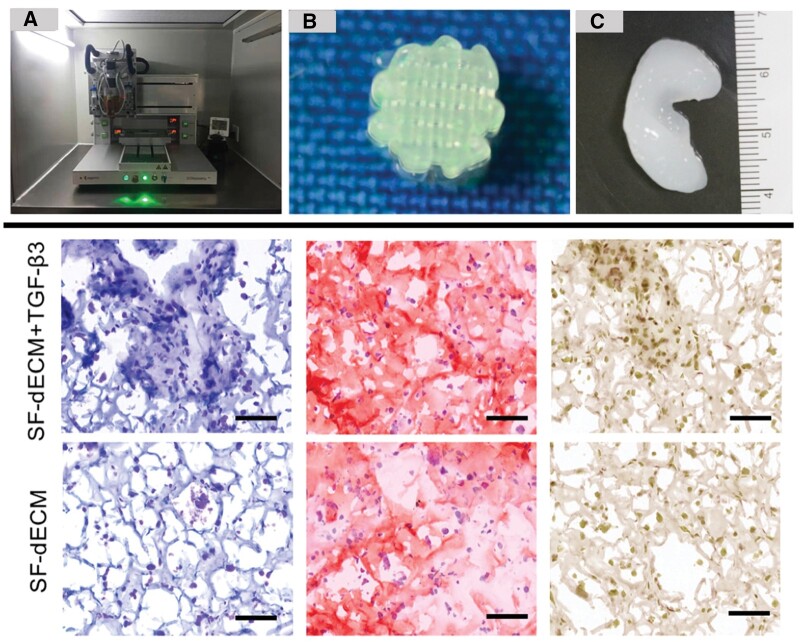
Chondrogenesis promotion of BMSCs after 28 days in decellularized ECM (dECM)-silk fibroin (SF) bioink doped with TGF-β3 for controlled release. Upper: 3D bioprinter used in the process (**A**); visualization of the printing procedure (**B**) and presentation of the resulting cylinder-like pattern (**C**). Lower: Histological sections of the printed constructs, stained with Masson’s trichrome (left) and safranin O (middle), along with immunohistochemical analysis (right) of collagen II, were conducted after 28 days of incubation. Scale bars represent 100 μm (reproduced with permission from [62]).

Without some method of crosslinking, either physical or chemical, most of tissue engineering scaffolds usually display fast degradation rate profiles. To negate this effect, crosslinking agents are often employed, which can mechanically reinforce the 3D structures but simultaneously can have a negative impact on the cytocompatibility status, as most of them are cytotoxic and small traces can still get entrapped inside the polymeric matrices, in spite of multiple washing cycles [139]. Silk fibroin protein is a natural biomaterial derived from *Bombyx mori* cocoons, which mainly consists of alternating beta-sheet crystallite domains, that lead to strong physical gelation without the need for further crosslinking. Moreover, silk fibroin is also water-soluble, it displays very high storage modulus values and medium proteolytic degradation rates in the presence of cells and great biocompatibility [140–142]. As such, it has been used a base material for the construction of cartilage promoting bioinks, without the use of any type of crosslinking [62] (Figure 2). On that note, Singh *et al.* [143] developed crosslinker-free bioinks comprising silk fibroin, gelatin and porcine chondrocytes (PCs), with the incorporation of gelatin aiming to provide further mechanical strength through the formation of interpenetrating polymeric networks, enhanced biocompatibility and low antigenicity levels. The presence of gelatin also facilitated a temperature-controlled phase transition, which significantly enhanced the printability of the bioink solution. After the optimization of the 3D printing parameters, two distinct structures, a small grid and a large 3D bioprinted ear were selected for the evaluation of the bioink attributes. The DSS analysis showed a very low dominant storage modulus at 1.5 kPa for the bioprinting solution, while the SEM imaging revealed a highly porous and grainy surface. The compressive modulus of the small grid scaffold and the 3D bioprinted ear was also evaluated at 100% strain, with the first peaking at 23 kPa and the second at 110 kPa, approximately. Additionally, the 3D printed ear swelling ratio reached to a plateau of an almost 10-fold mass increase compared to the scaffold’s initial mass after 15 h, while the degradation study was performed for the artificial ear structure, in the presence of simple PBS solution and a protease XIV solution, to better simulate the *in vivo* conditions. The results illustrated that in simple PBS, the construct showed a degradation rate of 10% after 30 days, but the addition of the protease XIV resulted in a staggering 60% degradation rate, at the same time point. In regards to its chondrogenic potential, the staining for GAGs and collagen depicted a significant increased effect between Days 1 and 14, with the PCR analysis for the COL-II, COL-X, SOX-9 and ACAN chondrogenic markers also presenting a steep upregulation profile between these two time points.

### Endothelium and cardiovascular bioprinting

Human organs, apart from some special cases such as cornea and cartilage, cannot sustain their structure and functionality solely depending on the diffusion of nutrients from the local environment. Thus, an intricate system of vascularization is usually essential to support the prosperity of the metabolically diverse organ systems [144]. Heart is a muscle which is inextricably bound to the vascularization system, since it acts as regulator for the blood flow inside the body and its pumping power dictates how the blood supply is distributed to the individual organs. Although there have been many advancements in the treatment of heart diseases, both in the field of surgical techniques [145] as well as in the field of pharmaceuticals [146], there is a great interest from the research community in investing efforts towards the development of new, less invasive technologies that, compared to the aforementioned methods, do not pose any significant threat of provoking adverse complications such as post-surgical bleeding, perforation or damage to the adjacent tissues or the jeopardization of the homeostasis of various physiological pathways due the release of toxic byproducts from the respective drug molecules. In addition, heart cannot regenerate automatically after it suffers critical damage and can often formulate scar tissue after surgical incision, leading to the impairment of the electrical conductivity in the tissue, resulting in arrhythmias and other heart related pathophysiologies [147, 148].

Stem cells compose a widely utilized cell model system in regenerative medicine applications since they can be isolated from different organs and possess the ability of self-renewal and differentiation towards multiple lineages, under appropriate exogenous probing [149, 150]. A number of recent studies have indicated that tissue engineering and cell injection strategies can have a positive impact on the cardiac function [151, 152], therefore making their intertwining a promising therapeutic avenue. In tissue engineering, one of the principal aspects that needs to be taken into consideration, is the ability of the various scaffolds to allow for adequate perfusion so that the necessary components can move through the artificial tissue structure and enable sufficient cell growth and proliferation [60, 144]. Many scaffolds designed for this purpose fail to meet the requirements for this complex architecture, leading to collapse under *in vivo* conditions [153, 154]. As such, pre-vascularization and the amplification of this effect is one of the big hurdles that needs to be overcome, before a tissue engineering platform is deemed suitable for clinical applications [154]. 3D bioprinting offers a new, more refined solution to address issues that stem from the biomechanical requirements that the classic 3D printing technology imposes, by allowing the design of bioinks with desired geometry, stiffness and cell density. Lately, advancements have been made in both endothelial [155] and cardiovascular [156] bioprinting, with researchers striving to transmute the *in vitro* and *in vivo* results into transplants that are viable in medical practice. Since the cardiovascular system consists mainly of soft types of tissue with storage modulus averaging at several kPa [65], usually squishier, natural biopolymers are selected as the base constituents for such bioinks, sometimes containing biomolecules that can enhance the under investigation biological responses [10, 157]. In some cases, where the fine tuning of the mechanical properties cannot be attained by using exclusively polysaccharides, more robust, biocompatible synthetic polymers are also employed in the mixture [158, 159].

Gelatin and alginate are two biomaterials that have found extensive use in cardiovascular 3D bioprinting due to the excellent biocompatibility of the first and the lesser biocompatibility but great gelling capacity of the latter [160, 161]. For example, Roche *et al.* [162] combined gelatin and alginate to generate 3D bioprinted cardiac patches, with hierarchical distribution of the endothelial cell network. Alginate was used to increase the mechanical stability of the resulting bioink by providing carboxyl moieties capable of ionic crosslinking. To increase bioinks’ degradation rate and structural integrity, methacrylate groups (MA) were also introduced to the gelatin solution, in order to produce photocrosslinkable GelMA. Human coronary artery endothelial cells and human dermal fibroblasts (hDFs) cell suspensions were mixed with the GelMA/alginate blends, and were followingly physically and photo-crosslinked to formulate the bioinks. The two cell lines were investigated independently. Cell viability, which was assessed up to 28 days of culture, was conducted by staining the bioinks with calcein-AM, ethidium homodimer and Hoechst cell nucleus stain and showed high cell survivability and proliferation status, for both cell lines. The cells’ assembling and organization inside the patches were also examined by CD31 (PECAM-1) antibody staining, which is a highly expressed glycoprotein receptor in endothelial cells’ membrane acting as a mediator for intercellular interactions, and Hoechst staining after 28 days. The laser scanning confocal microscopy images validated that the formation of characteristic tubular structures resembling the native endothelium did occur, for both cell types. Moreover, the contractility of the patches was observed by video light microscopy. Despite the double crosslinking, over the course of the 4 weeks experiment, most of the bioinks broke down into lesser fragments but these smaller pieces still induced a portion of the expected contractile activity, indicating the bioink potential as cardiac patches. On another work, Maiullari *et al.* [163] also capitalizing on alginate and GelMA as the main components, focused on the construction of bioinks containing extracellular vesicles (EVs) which are known to promote cellular communication. The EVs were produced by human umbilical vein endothelial cells (HUVECs) which were cultivated under various conditions, complete M200 medium in normoxia (CM normoxia), complete M200 medium in hypoxia (CM hypoxia), serum-free M200 medium in normoxia (SM normoxia) and serum-free M200 medium in hypoxia (SM hypoxia). Both hypoxia conditions resulted in the formation of vesicles with wider diameter, with those stemming from SM hypoxia being the largest ones. This fact showcases the adaptative ability of the cells to harsh conditions, and being able to negate them by producing larger and more potent exosomes. Enzyme-linked immunosorbent protocol (ELISA) revealed that SM hypoxia group also had the highest expression of the molecule PIGF and receptor VEGF-R2, which are pivotal mediators of neovascularization, while CM hypoxia group showed an increase in the production of VEGF-A and activation of receptor VEGFR-1, which are also associated with endothelium formation. Moreover, the bioprinted constructs containing the vesicles were placed inside mice to monitor the levels of neovascularization after a period of 60 days. The histological evaluation showed great cell viability for all four conditions but the SM hypoxia group had the most prominent effect on the growth of endothelium-like junctions and branches in the adjacent area. The team also conducted a series of *in vitro* experiments by cultivating peripheral blood mononuclear cells (PBMCs) containing the endothelial progenitor cells (EPC) fraction, in the presence of the four bioink compositions, to understand their impact on the maturation of the EPCs. In the case of SM hypoxia group, immunofluorescence protocols showed an upregulation of endothelial markers such as CD31, VEGFR2 and CD45, while also illustrated the formation of tubular structures, resembling ensembles that are present in the endothelial tissue.

Another approach that has been followed in the cardiovascular 3D bioprinting field is the utilization of ECM deriving from specific cell lines, which can then be furtherly used as a building block for various bioink compositions. Scaffolds that are constructed from different biomaterials, usually cannot fully mimic the physiological biological cues that allow cardiomyocytes to grow and this can lead to potential compromise of the structural integrity of the neosynthesized tissue [63, 164]. On the contrary, ECM-based scaffolds encompass a complex ensemble of biomolecules that have been secreted by cells of a specific organ, and can direct tissue growth with great detail, especially due to their spatial distribution which closely resembles native tissue [165, 166].

**Figure 3.**
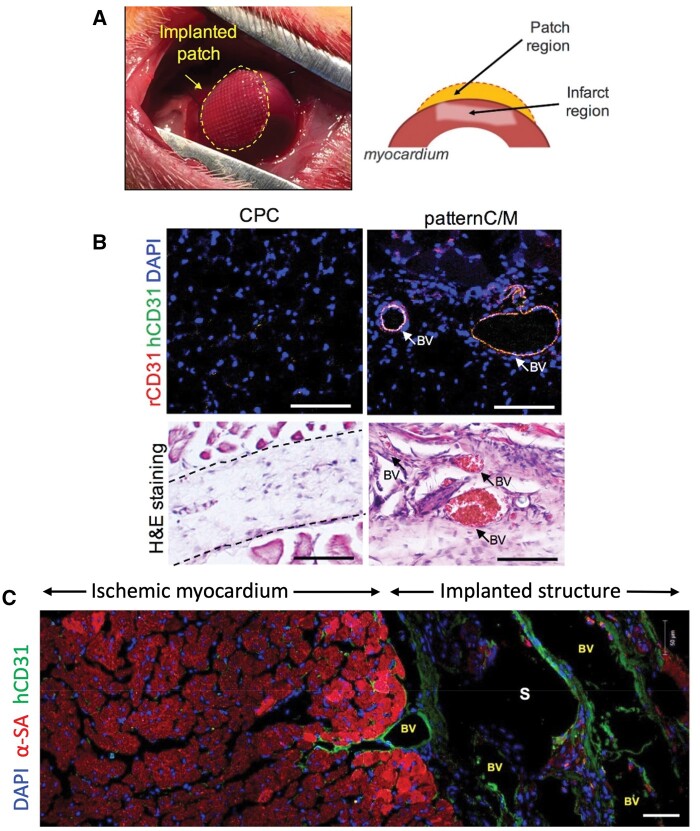
Bioprinted cardiac patch containing hdECM with VEGF and cardiac progenitor and mesenchymal stem cells for neovascularization towards the injured myocardium. Visualization of the implanted patch and schematic delineating the imaging area under consideration (**A**). Immunofluorescence staining and histological analysis 4 weeks after implantation (scale bars represent 200 μm) (**B**). observation of vessel formation within the pattern C/M patch and migration from the patch to the injured myocardium at Week 8 (scale bar represents 50 μm) (**C**). CPC is the patch using only cardiac progenitor cells (CPCs), pattern C/M refers to the fabricated patch by patterning CPCs and MSCs with VEGF alternatively, BV designates the newly formed blood vessel, S represents the supporting poly(ε-caprolactone) mesh and α-SA is α-sarcomeric actin (reproduced with permission from [170]).

Due to its nature, ECM-based 3D bioprinting has found applications in the construction of different cardiovascular medical devices [167, 168]. Bejleri *et al.* [169] for example, prepared 3D bioprinted cardiac patches by using ECM mixed with human pediatric cardiac progenitor cells (hCPCs) in order to be used as potential implants for restoration of myocardium functionality. Since ECM by itself cannot provide the necessary mechanical stability and printing viscosity, GelMA was also incorporated in the mixture which was photopolymerized to formulate the final bioink (ECM/GelMA). GelMA mixed with hCPCs without ECM bioink was used as control so that the impact of the ECM’s implementation could be illustrated. Cell viability was assessed at Days 1, 3 and 6 for the two compositions and the results were similar. A wide range of embryogenesis and myocardial differentiation markers were investigated by PCR including Connexin 43 (Cx43), GATA4 Myocyte enhancement factor 2C (MEF2C), β-myosin heavy chain (MYH7), vascular endothelial cadherin (VE-Cad), platelet endothelial cell adhesion molecule (CD31), vascular endothelial growth factor receptor 1 (FLT-1) and α-smooth muscle actin (ACTA 2). In all cases, except for GATA4, genes showed higher upregulation levels in the ECM/GelMA bioinks compared to the GelMA ones, underlying the effect of ECM on the cellular fate. Moreover, the supernatants of both compositions were collected and then used to feed HUVEC cell cultures, in order to identify the patches’ angiogenic capability. It was observed that when the conditioned media from the ECM/GelMA bioinks was administrated to the cell cultures, HUVECs acquired tubular formation at an almost double frequency compared to when fed with the supernatants from the GelMA ones. On the same note, Jang *et al.* [170] prepared a 3D pre-vascularized stem cell patch comprising ECM from heart tissue, hCPCs and mesenchymal stem cells (MSCs) blended with VEGF, and evaluated its cardiovascular properties compared to a collagen-based bioink, with the same cell populations (Figure 3). Polycaprolactone was used as a printing substrate to avoid construct collapse. Pictures from DAPI, actin and troponin staining revealed that the ECM containing bioinks had much more prominent effect on cells’ expansion and differentiation than the collagen ones. Also, by Day 5, the MSCs acquired the characteristic tubular formation of the endothelium structure, which is strongly indicative of the differentiation of MSCs towards endothelial tissue. The team performed a series of *in vivo* experiments by implanting the ECM bioink at the epicardium of mice that had previously suffered an induced myocardial infarction (MI), to monitor the patch’s regenerative capacity. The mice that received the transplant showed an excessive and irregular mass of tissue formation at the inflicted area compared to the non-treated MI control samples. ELISA analysis showcased elevated levels of endogenous VEGF production in the ECM bioinks compared to the control MI samples. Echocardiography was also employed to determine the cardiac contractility restoration of the ventricles. The examination of left ventricle showed an increase of contractility in the mice treated with the ECM bioink, validating the implant’s regenerative properties. In addition, after the surgical removal of the mice hearts, immunohistological assessment followed which pointed out that a big portion of the hCPCs had already differentiated towards cardiomyocytes but the sarcomeric organization was still not fully completed. Despite that, big areas residing on the opposite site of the heart trauma, were found to having expressed positive β-myosin heavy chain (βMHC) signals, which supports the group’s initial speculation that paracrine biological cues, even acting from distance, played a pivotal role in the cells’ assembling, proliferation and differentiation.

Although biomaterials have been widely utilized for the fabrication of various tissue engineering platforms, their use is usually associated with many drawbacks. More specifically, biomaterial-based scaffolds’ applications can be hindered by rapid degradation rates, suboptimal mechanical properties, toxic residuals during the preparation process, poor biological response and exhibition of strong immunogenic response [171]. An alternative approach that has been proposed is the synthesis of biomaterial free scaffolds, by mimicking the cell population ratios that are met in the native cardiovascular tissue and allowing them to produce their own ECM as supportive material [172, 173]. The scaffold-free technique has also emerged as a highly successful method for 3D bioprinting, for both endothelium and cardiac tissue growth. Yeung *et al.* [19] prepared a 3D bioprinted cardiac patch by combining three distinct cell types, including iPSCs, human adult ventricular CFs and HUVECs, in a 70:15:15 ratio. The cells were co-cultured in this proportion for a total of 3 days, till they formed spheroids whose formation was validated by immunochemistry protocols, and which were followingly used to 3D bioprint the cardiac patches. The 3D-printed constructs were furtherly cultured for 10 days before their *in vivo* implantation into mice. After 4 weeks, the patches were extracted from the mice and Masson trichrome staining was employed to determine the fibrotic areas post-surgery. Mice incised at the same region that the patch was implanted to but not having received the patch, acted as control. The trichrome staining indicated that the cardiac patch’s presence led to almost half the scar tissue formation compared to the control samples. Moreover, neovascularization process proved to be doubly efficient in the case of the mice that received the cardiac patch, as dictated by the staining of specific endothelial marker CD31. Scaffold-free bioprinting technology has also enabled the production of carefully tailored constructs that resemble the native artery and capillary structures [174, 175]. Norotte *et al.* [176], engineered small diameter grafts for vascularization by forming high density multicellular bioink mixtures comprising Chinese hamster ovary cells, human umbilical vein smooth muscle cells and human skin fibroblasts. FESEM microscopy was used to validate the formulation of the cellular spheres. To direct the printing of the multicellular building blocks into tubular structures, agarose rods were also printed individually, in various alternating motifs in regards to the printing pattern of the multicellular rods. This resulted in the construction of small-diameter multi-layered tubular vascular grafts, consisting of multicellular and agarose rods, with desired topology and spatial distribution of the cell spheroids. After 3 days in culture, the bioinks were stained for smooth muscle a-actin which is expressed in mature endothelium and Caspase-3 which is indicative of the levels of apoptosis. The staining showed the formation of expanded network of actin, while only a small portion of the initial cell number had committed to apoptotic state. Despite the innovative nature of the technique, building of the multicellular rods requires a great amount of cell spheroids, thus making the production of these bioinks quite costly and time consuming.

### Neural bioprinting

The prevalence of neurological diseases, as a result of either natural causes or by accidents, has taken a toll on the global health system, especially due to the limitations that rise from the application of traditional clinical treatment protocols [177, 178]. Neural tissue, based on topography, can be separated into the central and peripheral nervous system, with the central having negligible recovery properties and the peripheral one depicting only low levels of regeneration [179, 180]. Different cell lines have been employed as models for new medical research strategies, including neural stem cells (NSCs), mesenchymal stem cells (MSCs) Schwann cells, oligodendrocytes and even olfactory ensheathing cells [178, 181]. Neural tissue engineering scaffolds produced by 3D printing should exhibit excellent biocompatibility and cell proliferation [182, 183] as well as optimized fidelity and printability [184]. Moreover, in order to mimic the electricity flow that the physiological neurons allow for, biomaterials with electroconductive properties must be also integrated into the final construct [44, 185]. Most often, such materials have detrimental effects for the cell viability and the biodegradation rate of the resulting scaffolds, making their incorporation quite taxing [169].

In the recent years, research groups worldwide have shifted their focus away from classic 3D printing approaches to neural 3D bioprinting, in order to cope with the complexities that the intricate physiological neural networks demonstrate [186]. This technique enables the construction of structures with tailored geometry, mechanical properties and favorable biological responses. In the case of bioinks, cells are encapsulated inside the scaffolds, thus a great need for interior corridors and cavities formation must be satisfied so that they have the necessary space to grow and proliferate. To this end, neural bioinks have been prepared either by combining only natural biomaterials together [48] or by mixing natural and synthetic biopolymers to enhance the scaffolds’ mechanical stability or biochemical properties [187, 188].

Gelatin is one of the most commonly used proteins for neural bioprinting since it possesses excellent biocompatibility due to its RGD sequences (arginine, glycine, aspartic acid) which enhance cells’ adhesion to the artificial matrix [189] and a soft texture which is ideal for neural biomimicking applications. One disadvantage of gelatin is its inability to form firm gels at 37°C and its rapid degradation under *in vitro* cultivation conditions or inside the human body. For this reason, it is often combined with other biomaterials to produce more robust scaffolds [48]. Wu *et al.* [51] fabricated neural bioinks with Schwann cells by preparing a blend of gelatin and alginate with the aim to examine their biological response under *in vitro* and *in vivo* conditions. The secretion level of a series of different neurotrophic factors was investigated through quantitative real-time polymerase chain reaction (qRT-PCR), including nerve growth factor (NGF), brain-derived neurotrophic factor, glial-derived neurotrophic factor (GDNF) and platelet-derived growth factor (PDGF). An ELISA kit for NGF was also employed as a supplementary method of determining NGF release from the cells. Scaffolds depicted a greater viability status as well as higher levels of growth factors secretion compared to the 2D control counterparts, validating the role of bioinks in enhancing the neural growth process. S100B calcium binding protein B staining was also used to detect the levels of this universal neural protein, which is indicative for cell survival, which also demonstrated great results for the bioinks. In regards to the *in vivo* experiments, the bioprinted constructs were placed subcutaneously in mice and after 4 weeks they were removed to examine their biological status and mechanical integrity. Although the scaffolds had turned quite loose due to the rapid displacement of the Ca^+2^ which was used to crosslink the alginate carboxyl groups, they exhibited great cell proliferation and survivability.

In order to counter such fast degradation rates, gelatin can be chemically modified to introduce groups that enable covalent crosslinking. One such modification is the integration of MA which can be employed for photopolymerization techniques through using a photoinitiator [160, 184]. Realizing the pivotal role of growth factors for the development of all tissues, research groups have tried to encapsulate and deliver them at controlled rates which mimic the *in vivo* conditions [190, 191]. Chen *et al.* [192] prepared a modular bioink based on GelMA, which combined microspheres containing NGF and GelMA scaffolds. Firstly, GelMA was prepared from gelatin following an established protocol and was allowed to mix with chitosan under mild acidic environment. Subsequently, microspheres of GelMA/chitosan were formed by employing a microfluidic approach which were then crosslinked by using Irgacure 2959 photoinitiator to bind the GelMA molecules and to create hydrogel microspheres. The mechanical properties of the microspheres were also evaluated, with values of *G*′ ranging between 17 and 35 Pa and *G*″, 0.7–2,2 Pa, being close to those of the native neural tissue. PC12 cells from rat pheochromocytoma were seeded for 1 day unto the microspheres and then were mixed with a GelMA bioink containing RSC96 Schwann cells to formulate the final bioink. The idea behind the co-culture was based on the fact that Schwann cells secrete a lot of different neural growth modulating biomolecules which can in turn affect the differentiation of the PC12 cells under co-culture conditions. Two different bioinks were prepared, one encapsulating NGF inside the GelMA/chitosan microspheres and one without, to compare the effect of NGF release on the bioink biological response. The differences between the viability of the two bioprinted constructs was negligible, but those containing NGF exhibited much longer axon length for the neurites, implying that the differentiation process took place more efficiently in comparison to the bioinks not containing NGF.

The electroconductivity of a neural bioink is also a very important parameter for the biomimicking of the flow of electricity that the native neurons depict. Song *et al.* [58] tried to create bioinks with enhanced electrical conductivity, while retaining good biological response. Poly (3,4-ethylene dioxythiophene)/poly(styrene sulfonate) (PEDOT/PSS) was selected as the electroconductive reagent due to its biocompatible nature. Although PEDOT by itself is electroconductive, it is not hydrophilic, so it is usually combined with PSS to increase its water solubility. Since these two materials are not biodegradable, in this work, chondroitin methacryloyl (CMA) was used to increase the biocompatibility/biodegradation profile of the bioinks. The introduction of CMA though hinders the charge transport. This was overcome by using tannic acid as a secondary dopant via redox polymerization. In total, three different bioinks were fabricated using NSCs, GelMA/PEGDA/CMA/PEDOT/PSS (GPP), GelMA/PEGDA/CMA/PEDOT/PSS with tannic acid (GPP-TA) and GelMA/PEGDA acting as control (GP). Bioinks were produced by photopolymerization. All compositions showed great cell growth and proliferation status between Days 1 and 7 and rheologically similar profiles, with *G*′ ranging approximately between 150 and 250 Pa, validating the bioink mechanically soft nature. What is interesting to note is that although the addition of PEDOT/PSS did lead to an increase of the electrical conductivity, the presence of tannic acid did not seem to affect this property significantly. Staining for beta-tubulin III (Tuj-1), a type of tubulin that regulates intracellular/axonal transport and structure maintenance and glial fibrillary acidic protein (GFAP) which is representative of the metabolic activity of glial cells revealed that the GPP-TA bioinks had higher expression of Tuj-1 and lower expression of GFAP compared to the GPP ones, which showcases the capability of the GPP-TA composition to promote neuronal rather than astrocytic differentiation. Finally, the neurites formed in GPP-TA also demonstrated longer neurites, reinforcing the validity of the staining findings.

**Figure 4.**
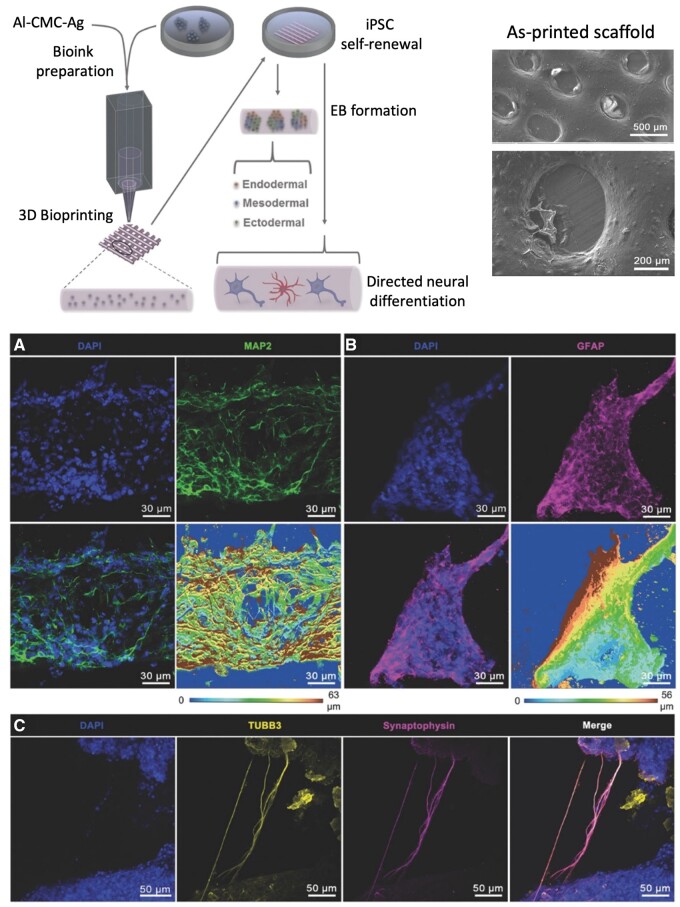
Schematic of neural tissue bioprinting employed for the 3D culture and differentiation of induced pluripotent stem cells (iPSCs) (upper left). Scanning electron microscopy (SEM) images depicting the as-printed scaffold and the incorporation of iPSCs within the structure (upper right). Al-CMC-Ag is a hydrogel blend of alginate (Al), carboxymethyl chitosan (CMC) and agarose (Ag).

Another soft natural polysaccharide with a wide array of biological applications is chitosan, due to its biocompatible nature and antibacterial properties [193]. These traits are mainly derived from the presence of its amino groups and its many hydroxyl groups. A downside of chitosan is that it requires acidic pH in order to dissolve properly, restricting its application as a bioink component. This hurdle can be surpassed by modifying chitosan with the introduction of water-soluble groups. One of the most common routes followed is the introduction of carboxymethyl groups to its amino and hydroxyl groups, resulting in the formation of N, O-carboxymethyl chitosan (NOCC) [194, 195]. Gu *et al.* [196] prepared a bioink comprising alginate, carboxymethyl chitosan and agarose loaded with iPSCs. This work revolved around the formation of self-organizing embryoids inside the bioinks, in order to mimic the initial cell gatherings that take place during the early embryonic development. To this end, a series of differentiation markers for three different cell lineages, endoderm, mesoderm and ectoderm were determined by qRT-PCR. The bioinks demonstrated excellent cell viability and the formation of neurites was evident, even by Day 9. OCT4 multipotency factor showed downregulation which confirms the stem cells’ maturation to neural having already occurred. Homeobox genes NANOG, TDGF1, UTF1, H19 and PDX1 which are markers for endodermal, Hand-1 and IGF-2 for mesodermal and finally NES and TUBB-3 for ectodermal, were all upregulated compared to the 2D control (Figure 4).

Immunophenotyping analysis conducted 40 days post-printing, encompassing 30–37 days of neural induction and differentiation (A–C). Cells stained with DAPI and expressing the pan-neuronal marker MAP2, revealing neural processes extending throughout the constructs (A). Immunofluorescence staining for the radial glia and astrocyte marker GFAP (glial fibrillary acidic protein), displayed in depth-coded images along the *Z*-axis (0–63 and 0–56 µm, respectively), highlighting the spatial arrangement of cells within the constructs (B). Cells stained with DAPI and the pan-neuronal marker TUBB3 (tubulin beta 3), indicating the presence of neuronal cell clusters, synaptophysin colocalized with TUBB3-labeled processes, indicating the formation of synaptic connections between neuronal cells (C) (reproduced with permission from [196]).

Polyurethane is a synthetic polymer which is used as a base component in innumerous everyday life products, such as foams and different types of plastics. Since it depicts low levels of biocompatibility and no biodegradability, it needs to be modified in order to accommodate the attributes that a bioink must possess. Modified polyurethanes have proven to demonstrate great cellular response, in regards to both cell proliferation and differentiation [61, 197]. Huang *et al.* [198] prepared modified and biodegradable thermoresponsive waterborne polyurethanes, mixed with two types of graphene, in order to create 3D platforms that can induce the differentiation of NSCs. In more detail, the modified polyurethane consisted of two distinct biocompatible synthetic polymers, poly(e-caprolactone) diol (PCL diol) and poly(d, l-lactide) diol (PDLLA diol). Following an established protocol, graphene platelets and GOs were prepared and physicochemically characterized. In the case of the graphene platelets, pluronic P123 was employed to stabilize them in the water solution. Subsequently, the polyurethanes were mixed with the graphene platelets/pluronic and the GOs, resulting in (i) polyurethane/graphene platelets/pluronic (PU/G-P) and (ii) PU/GOs, respectively. No graphene containing polyurethanes (PU) were used as control. The rheological analysis showcased that the addition of graphene led to a decrease in the mechanical strength of the hydrogels compared to the control PU, bringing the *G*′ closer to 1k Pa which is considered ideal for neural tissue engineering applications. Cytotoxicity of the bioinks was evaluated for a total of 14 days, with the PU/G-P and the PU/GO compositions surpassing the PU ones by a large margin, and the PU/G-P retaining the highest values at all time points. To assess the NSCs differentiation potential in the presence of the bioinks, qRT-PCR was conducted for three markers, nestin, β-tubulin and GFAP. Nestin levels were higher in PU compositions, indicating that the differentiation was lower in comparison to the PU/G-P and the PU/GO ones, while the PU/G-P bioinks exhibited the highest values for both β-tubulin and GFAP. These results coincide with the immunofluorescence staining findings for the same markers, proving the superiority of the PU/G-P and the impact that the graphene can have on the differentiation capacity of the NSCs. Modified polyurethane based bioinks have also been evaluated under *in vivo* conditions. Hsieh *et al.* [199] constructed a series of water-soluble biocompatible polyurethanes from different aqueous dispersions of polyurethane nanoparticles, capable of forming hydrogel upon heating. Four different compositions were prepared, 30% PU1, 25% PU1, 30% PU2 and 25% PU2, with the % number representing the percentage of the solid content of polyurethane nanoparticles dispersion and the PU1/PU2, the chemistry of the polyurethane soft segment. NSCs viability was assessed for 24 and 72 h, with the 24 h timepoint presenting no significant differences but at 72 h, the PU1 compositions depicted a decline in cell number while the PU2 a sharp increase, with the 25% PU2 exceeding the 30% PU2. The neuronal-related markers nestin, β-tubulin and MAP2, demonstrated higher expression in the PU2 compositions, while the 25% PU2 samples exhibited the lowest levels of the glial GFAP marker, showing the differentiation specificity of that concentration towards the neurite rather than the glial cell line. Moreover, among the different neurotrophic factors evaluated through RT-PCR, only NGF demonstrated a higher expression in the PU2 bioinks compared to the PU1 ones. The *in vivo* experiments were conducted by examining the spontaneous coiling contraction of zebrafish embryos, which is an intrinsic reflex associated with normal neuronal development. This physiological process was exogenously impaired by the addition of 2% ethanol and the abnormal embryos were followingly treated with cell-laden bioinks to validate how much of the initial activity of spontaneous coiling contraction is recoverable. Although ∼50% of the embryos treated with both the PU1 concentrations showed only slight recovery rate of the physiological contraction, the insertion of the 30% PU2 and 25% PU2 bioinks led to a full recovery of ∼65% and 70% of the total treated embryos, respectively, signifying the overall superiority of the 25% PU2 composition as a neural bioink.

### Skin bioprinting

Skin is the largest human organ, acting as a soft and simultaneously elastic barrier whose primary role is the separation of the internal body from the external environment. Its multilayer structure provides mechanical protection against external stimuli, obstruction of pathogenic microorganisms invasion and regulation of homeostasis related processes. Skin tissue layers can be broadly classified in three main categories: (i) the first layer known as epidermis hosts a number of different cell types such as keratinocytes, melanocytes, Langerhans and Merkel cells, constituting the first line of defense but with limited mechanical stability, (ii) the intermediate layer known as dermis, which can be divided into the papillary outer portion that comprises a loose interpenetrating network of fibrils and the reticular matrix that retains much thicker fibril formulations, thus resulting in enhanced mechanical tensile strength and (iii) the outmost inner layer designated as hypodermis, which is rich in lipid cells that are glued together by the mediation of small ECM isles [200].

Skin ECM consists of a multitude of biological macromolecules that have both structural and biofunctional role, among them being various collagen types, GAGs, proteoglycans, fibrillins, laminins and integrins [201]. Genetic mutations that target these substrates are quite common in human population, such as Ehlers Danlos [202], Marfan [203] and epidermolysis bullosa syndrome [204]. During the healing process, the main responsibility of skin is the protection of the whole body, thus restoration of skin barrier precedes the reconstruction of compartments of dermal ECM, which endow its mechanical properties. Re-epithelialization predates the *de novo* formation of dermal tissue [205, 206]. Additionally, the deposition of ECM in the renewal phase should take place gradually, following particular patterns, allowing for the replenishment of the damaged tissue and concurrently ensure sufficient spatial interconnectivity of the distinct reformed layers [207].

Based on the features described above, the growth of human tissue skin analogues dictates the development of hyper complex biomimicking matrices. There have been attempts to utilize 3D bioprinting technologies to prepare such constructs, by employing different ingredients of native skin such as collagen [208], gelatin [209], fibrinogen [210] and even through biomaterials free approaches [211]. Following a sophisticated methodology that couples inkjet bioprinting with integrated imaging technology, Albanna *et al.* [212] reported on the production of bioinks consisting of fibrin/collagen, combined with dermal fibroblasts and epidermal keratinocytes for the *in situ* replacement of extended skin trauma. The resulting multi-layered scaffolds showed to exhibit accelerated wound closure, reduced inflammation process and rapid re-epithelialization, displaying great promise as a potential on-site therapeutical avenue for large skin defects.

Although all these natural biomaterials strongly mimic the native skin architecture, their contribution to mechanical integrity and elasticity is tied to the formation of dense interpenetrating networks present in human skin layers, as singularly they lack the necessary stiffness required to replicate the physiological skin flexibility. As such, most of the 3D bioprinted skin implants, although depicting great biological effect, they present reduced mechanical efficacy. To mitigate this downside, various crosslinking methodologies have been proposed [213–215]. An interesting crosslinking method without bearing any detrimental effect for cell viability, is the use of tyrosinase, a native enzyme which is able to spontaneously oxidize various tyrosine residues present in most of skin proteins, leading to the formation of *o*-quinone moieties which in turn can react with available amine groups through Schiff base reaction [216]. Capitalizing on this chemistry, Shi *et al.* [217] prepared bioinks designed for dermis/epidermidis reconstruction, after the mixing of collagen, GelMA, tyrosinase (Ty) and Irgacure 2959 with three cell types, human melanocytes (HEM), human keratinocytes (HaCat) and hDFs). The bioinks underwent double crosslinking, both due to Ty covalent bonding of amino and *o*-quinone groups present in collagen and GelMA, as well as through photopolymerization of GelMA at 365 nm UV light for 40 s. As expected, rheological analysis of the bioink blends revealed a rise in viscosity values as Ty content increased, confirming the occurrence of tyrosinase crosslinking. Cell viability was assessed after 1, 4, 6 and 14 days. All bioinks exhibited high cytocompatibility with no apparent variations in regards to Ty concentration. Proliferation and scratch assays showed a strong correlation of Ty to enhanced proliferation for HEMs and simultaneous inhibition of HDFs migration, a positive outcome since over-growth of HDFs can lead to irregular ECM deposition and longtime scarring. Bioinks with and without Ty were subcutaneously inserted in the back of rats, and wound closure analysis together with histological evaluation were performed after 7, 14 and 21 days. Physiological healing without the mediation of any implants was used as control condition. Results showed that up to Day 7, the two bioinks evoked more rapid regeneration than the control, and by Day 14, all conditions showcased full wound closure. Interestingly, on Day 21, the thickness of the newly formulated epidermidis was found to be much thicker in case of the two bioinks, compared to the control, signifying the potent nature of these bioinks as skin implants.

**Figure 5.**
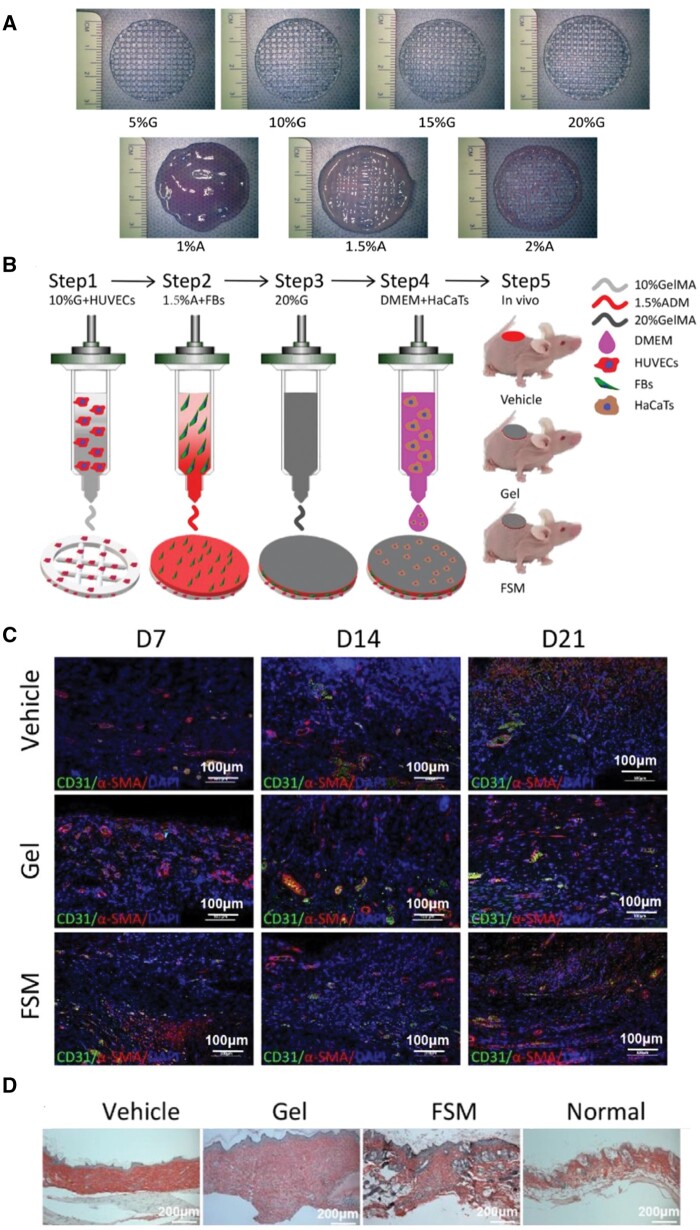
3D bioprinted skin constructs consisting of gelatin methacrylate (GelMA, G) and acellular dermal matrix (ADM, **A**), at various concentrations, to determine optimal printing fidelity (A). Schematic representation of three layered bioink assembly process, with the bottom bioink being 10% wt GelMA mixed with HUVECs acting as vasculature mesh, middle layer consisting of 1.5% wt ADM and fibroblasts as the dermis analogue and the third layer comprising of 3D printed 20% wt GelMA seeded with human keratinocytes (HaCaTs), designated as the epidermis layer; FB, fibroblasts (**B**). Confocal images for CD31/α-SMA angiogenic markers (**C**) and sirius red collagen staining from optical microscopy for the three bioink types (**D**), over a period of 21 days; α-SMA, α-smooth muscle actin; FSM, functional skin model; gel, GelMA; vehicle: 1.5% ADM (reproduced with permission from [218]).

Since most of the more severe lesions of a physiological trauma affect more than one skin layer, it is crucial for a skin graft to be able to facilitate multi-faceted regeneration, including *de novo* re-epithelialization of the region. Jin *et al.* [218] reported on the production of multi layered structured bioinks with full thickness skin properties and a fully developed vascularization network to their support. To achieve this, the team fabricated two different bioinks, one on top of the other. The first layer comprised GelMA loaded with HUVECs, acting as the vasculature mesh, while the second one contained acellular dermal matrix (ADM) blended with fibroblasts, designed for the restoration of the dermis section. Selection of ADM was favored as bioink component due to its enriched nature with cytokines that are necessary for skin cell growth and differentiation. On top of the second layer, a 3D printed scaffold of GelMA was prepared as the epidermis patch, which was subsequently seeded, and not bioprinted, with HaCat cells, as it has been shown that keratinocytes display enhanced adhesion and proliferation on stiffer substrates [219]. This sandwich like formation reduced the exposure of the more brittle ADM mid layer to mechanical stresses, confining it between the GelMA bioinks. Two bioinks, one with and one without the third HaCat seeded cap were inserted subcutaneously in mice for *in vivo* assessment. Both gap of wound and histological analysis revealed that the three layered bioink significantly quickened the healing process, over the two layered one and the non-treated control counterpart. Confocal staining for CD31 and α-SMA angiogenic markers showcased after 21 days that the three layered bioink retained the highest number of blood vessels per square mm. Additionally, Sirius red collagen optical staining demonstrated that both bioinks had much more pronounced collagen production compared to the control condition. Figure 5 demonstrates some of the aforementioned key elements of this study.

Excretion of sweat from sweat glands dictates a pivotal role in many of the skin physiological processes, such as regulation of temperature, humidity control and removal of toxic byproducts whose accumulation could prove harmful towards healthy tissue. Sweat glands are broadly divided into two groups, the eccrine ones which are appendages closer to skin surface that chiefly revolve around homeostatic thermoregulation, and the apocrine ones that communicate with hair follicles and are responsible for the production of bacteria enriched sweat, in areas such as the head, armpits and perineum [220]. When extended skin trauma occurs, together with the three basic skin layers, hair follicles and sweat glands can also be severely damaged, sometimes irreparably. Hence, new 3D bioprinting techniques have shed new light on the reconstruction of such modalities [221, 222]. Liu *et al.* [223] investigated the role of porosity of ECM in the maturation of epithelial progenitor cells (EPs) towards sweat glands. They prepared four different bioinks consisting of gelatin, alginate and EPs, with and without the inclusion of plantar dermis (PD) and by using two different nozzles, 300 and 400 μm, to achieve different porosity degrees. PD contains many dermal components, including a vast collection of ECM proteins and growth factors such as BMP-4, whose bioactivity has been associated with dermal and sweat gland differentiation. Constructs were physically crosslinked with the use of CaCl_2_ solution. All bioinks presented good printability, with the ones produced with the 300 μm nozzle being less bulky, thus resulting in larger pores between the printed strains. Cell viability of EPs was examined after 1, 7 and 14 days of culture. No significant differences could be detected, with cell number increasing between each subsequent time point. Release kinetics of BMP-4 as a result of gelatin degradation was also evaluated, with the 300 μm nozzle bioinks showing a faster release rate compared to the 400 μm condition, as expected, due to the bigger width of the bioprinted lines in the latter case. Finally, sweat glands luminal epithelial markers K18 and K19 expression was investigated through confocal imaging after 5 days. Out of the four bioinks, the 300 μm doped with PD expressed only K18, while the 400 μm doped with PD showed upregulation of both markers, underlining that smaller porosity can impact the sweat gland differentiation process. Conversely, no PD containing counterparts had zero expression.

### Bioprinting of liver, kidney, pancreas and lung

**Figure 6.**
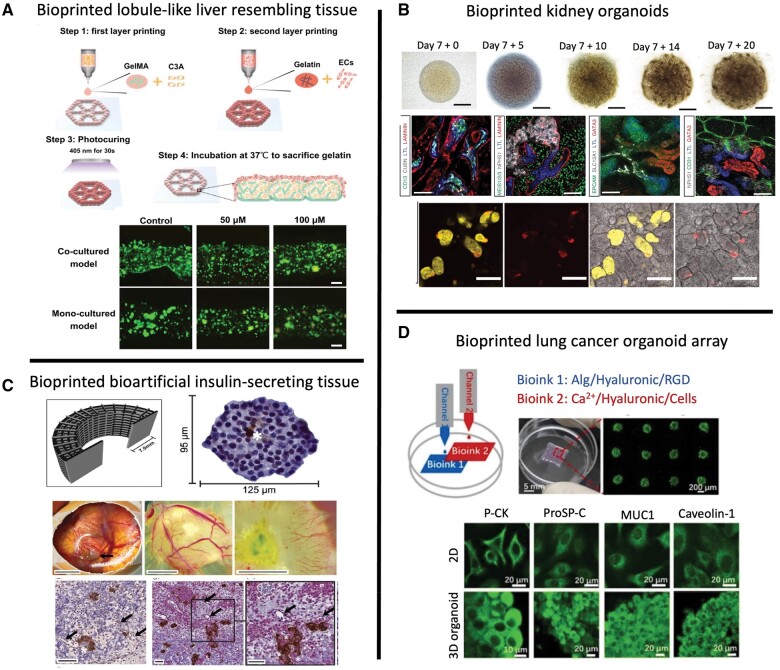
Representative images for organoid bioprinting. Co-culture of C3A and HUVECs bioprinted in GelMA and gelatin hydrogels into lobule-like structures resembling liver tissue for evaluation of the drug sorafenib in co-culture liver cancer and mono-culture liver models (**A**, top left). Schematic illustrations outlining the process of bioprinting endothelialized liver lobule-like constructs in four steps (upper panel). Live/dead assay images depicting various 3D liver cancer models after incubation with different concentrations of sorafenib (lower panel) (reproduced with permission from [231]).

Liver is an organ committed to a wide array of biological functions, extending from the production of various proteins and other macromolecules associated with metabolism, storage of energy in the form of glycogen, metabolism of fats, activation of specified enzymes and being the center for the detoxification of drugs and toxic metabolic byproducts [224]. To distribute all its endogenously produced biomolecules throughout the human body, as well as to provide a pathway for the removal of waste products, the presence of a pervading blood circulatory system is mandatory. At its core, liver is composed of small, vascularized lobes that encompass a stunningly large ensemble of distinct cell types, making any attempts to biomimick them a rather challenging task [55, 64, 225]. However, liver is an organ prone to exhibit various pathophysiologies [226, 227], thus necessitating the development of new efficacious regenerative medicine techniques that can successfully restore its full functionality. There have been different attempts through bioprinting aiming for the reconstitution of liver tissue [228, 229] or towards the refinement of methodologies for drug screening applications [230, 231] (Figure 6A). Goulart *et al.* [232], evaluated the role that the human iPSCs’ dispersity has inside a bioink matrix on their differentiation potential towards mature epithelial cells. To investigate this hypothesis, the authors reported on the preparation of two bioink types regarding cell dispersity, single cell dispersion of iPSCs (SCs) and sphere-like bioinks (SPs) containing iPSCs in aggregate formation. For both bioinks, an alginate/pluronic F-127 blend crosslinked in CaCl_2_ solution served as the scaffolds’ matrix. Both bioinks were extrudable at pressures lower than 60 kPa, with the bioprinting process thus imposing minimal stress to the cells during the extrusion phase. Viability of the SCs and SPs was assessed through live/dead assay, revealing a decline pattern to 60% and 80%, respectively, after 18 days, compared to Day 1. In regards to their proteome profile, both bioinks shared similar statuses, without significant differences. RT-qPCR after 18 days showcased a significant upregulation for the SPs for a series of hepatic phase I related enzymes including CYP3A4, CYP1A2 and CYP1A1, compared to SCs, being indicative of the stronger hepatogenic effect of the first ones. Also, CDH1 and CDH2 markers, which are associated with the retaining of the mesenchymal phenotype for the iPSCs when upregulated, were found to be more prominently expressed in the case of the SCs, rather than the SPs. These results demonstrate how cell–cell interactions within the confined subareas of bioink internal cavities affect cell survival and differentiation potential.

Kidneys belong to the retroperitoneum space, with their main role being the filtration of the circulating blood and the formation of urine, with the latter’s consistency depending on the removal and reabsorption processes of multiple molecules constantly taking place in different sections of the kidneys. These substances can vary in nature, including proteins, ions, drugs and polysaccharides [233, 234]. As such, kidneys are responsible for pH maintenance, removal of organic and non-organic toxic byproducts and regulation of arterial pressure. There is a large variety of risk factors that have been linked to the hampering of kidneys’ physiological role leading to chronic renal diseases, among them being hypertension, diabetes Type 1, obesity and cancer [235–237]. Current therapeutic approaches for patients with end-stage renal diseases constitute mainly of dialysis or organ transplantation methods, both accompanied by medical intervention practices with high risks for the patients, such as contaminations, long transplant waitlists and possibility of transplant rejection. 3D bioprinting has been described to produce constructs that can support renal tissue regeneration, with only a finite number of publications having focused on this topic. Researchers have utilized biomaterial-free approaches to develop whole kidney tissue analogs [238] (Figure 6B), while others have employed KdECM (kidney decellularized extracellular derived matrix) from organic kidney tissue to develop bioprinted constructs for tissue engineering applications [239, 240]. In one such work, Ali *et al.* [241], aiming to fabricate a potent renal microenvironment, prepared photopolymerizable KdECMMA (kidney methacrylated decellularized ECM) containing bioinks, by mixing it with a blend of HA, gelatin, glycerol and human primary kidney cells (hPKCs). To obtain the KdECMMA, renal dECM was harvested from porcine kidneys, which was subsequently subjected to dissolution, methacrylation and multiple washing cycles, to purify it from unreacted moieties. Surprisingly, KdECMMA growth factor and cytokines content was found to be significantly enriched compared to that of pure KdECM, except for prokineticin-1, GDNF, and PDGF-AA which were absent in the KdECMMA, despite all the modification steps. Among the three different concentrations of KdECMMA that were used (10, 20 and 30 mg/ml), only the 30 mg/ml KdECMMA containing scaffolds managed to retain their structure after photopolymerization for more than 2 weeks, however depicting noteworthy deformation. Young modulus of the photopolymerized KdECMMA scaffolds ranged between 2 and 4 kPa, for the 10 mg/ml and 30 mg/ml concentration respectively, implying that the resulting scaffolds were quite soft. The cell viability of the three KdECMMA bioinks was also compared to that of KdECM and GelMA bioinks through live/dead staining after 1, 3 and 5 days of culture. All scaffolds displayed an increase in cell number between the subsequently examined time points, with the KdECMMA having the highest cytocompatibility level. To evaluate the sodium reabsorbance levels of hPKCs, which is a vital characteristic of the activity of proximal tubular kidney cells, sodium green fluorescence imaging was conducted after 14 days of culture. The images revealed significant higher sodium ions concentration in the case of kdECMMA bioinks compared to GelMA ones, thus indicating higher levels of activity of the sodium/hydrogen exchanger. Additionally, histological analysis after of 14 days for tubular marker AQP1 and glomerular marker NPHS2 was executed. Results showcased that the hPKCs inside the KdECMMA bioinks adopted the tubular and glomerular kidney-specific cell morphologies at a greater frequency compared to control GelMA scaffolds, further supporting their suitability as renal tissue reconstitution analogs.

Pancreas is a long abdominal organ, primarily dedicated to the production of hormones and enzymes responsible for food digestion and energy storage. Its head region comes into direct contact with duodenum, providing a release gateway for all its products towards the gastrointestinal tract, where a significant part of food digestion takes place. Insulin is a hormone that is secreted from pancreatic β-cells, acting as one of the main regulators for the uptake of glucose from blood to be furtherly used as a building block for the construction of different biomolecules, including glycogen, the main polysaccharide responsible for cells’ energy reserves [242]. Diabetes is one of the most prominently established and investigated metabolism related pathology, which is the result of either lack of insulin secretion from β-cells (Type 1) or due to β-cells’ reduced sensitivity towards the hormone (Type 2), which in both cases lead to abnormal levels of glucose in circulating blood [243]. 3D bioprinting has been used to fabricate structures that can boost insulin production from β-cells, in order to attenuate the negative effects of diabetes, especially Type 1 [244–246] (Figure 6C). One efficient method of producing bioinks with enhanced insulin secretion is the implementation of whole pancreatic islets derived from native pancreas, which include large concentrations of β-cells [247, 248]. Capitalizing on this technique, Wang *et al.* [249] worked on the 3D bioprinting of whole pancreatic islets mixed with a HAMA/pancreatic extracellular matrix (pECM) blend and investigated how the engulfed cells’ response varied compared to that of 2D conventional pancreatic islet control cultures, in regards to different aspects of β-cells physiological routine such as insulin production and specific pancreatic markers’ upregulation. The pECM was isolated from native porcine pancreas and was mixed with HAMA in different concentrations, ranging from 0 to 20 mg/ml, together with whole pancreatic islets. The bioprinted constructs were then photopolymerized at 405 nm with the mediation of lithium phenyl 2,4,6 trimethylbenzoylphosphinate (LAP). The Young modulus of the scaffolds was found to be between 4 and 8 kPa for the pure HAMA and the 10 mg/ml pECM bioinks, respectively, while the 20 mg/ml concentration displayed, surprisingly, slightly lower values than the 10 mg/ml one, close to 7 kPa. Cell viability at Days 3, 5 and 7 had comparable levels for all concentrations of pECM and pure HAMA scaffolds, showcasing a growth pattern between subsequent time points. Insulin secretion was examined after 70 min, with the 20 mg/ml pECM bioinks retaining significantly higher hormone production than the rest, findings that were also validated by insulin confocal staining images. Additionally, the 20 mg/ml pECM and the HAMA scaffolds were transplanted into diabetic mice and an intraperitoneal glucose tolerance test after 100 days followed, to further monitor the blood glucose levels in the animals. The pECM bioinks had significantly lower glucose levels than the HAMA ones, which stands compliant with the *in vitro* results, with both of them being indicative that the presence of pECM had a positive influence on insulin boosting process. qRT-PCR was also conducted for pancreas islet-related genes of E-cadherin, N-cadherin, fibronectin, laminin and Rac1, which all retained the highest upregulation status in the case of the 20 mg/ml pECM composition. Finally, the *in vivo* endothelization capacity of the two bioinks was assessed by the additional implementation of HUVECs into the cellular mixture and their transplantation into mice for 3 months. As expected, GFRA2, ACTA2, ANGPT2 and PECAM1 vascularization related markers were found to be upregulated in pECM bioinks through qRT-PCR, providing further insight for the neovascularization capabilities of pECM.

Lungs are the organs responsible for breathing, which can be broadly broken down into two major phases, the inhalation stage during which the thoracic pressure lowers as a result of the equivalent muscle groups’ contraction, allowing air to flow into the lung parenchyma, and the exhalation phase that constitutes the reverse process. During inhalation, fresh atmospheric oxygen enters particular spaces of lung parenchyma organized by pulmonary alveoli cells and is then transferred through the vasculature system towards the different organic systems of human body to be utilized for cellular metabolic reactions. As a result of these processes, carbon dioxide is produced, which travels back to the lungs and is excreted through exhalation [250]. Lung tissue displays an astonishingly large variety of pathologies, including metabolism-related syndromes, susceptibility to different microbial loads, as well as autoimmune and neoplastic diseases [250–253]. 3D bioprinting methodologies have enabled the development of the fabrication of alveolar lung biomimicking scaffolds [254, 255] but also lung cancer imitating models, to better clarify the molecular pathways involved in their causation [256, 257]. In particular, 3D bioprinting has also emerged as a promising methodology for drug delivery that aims either for lung tissue reconstitution or for the production of vessels that can act as potent drug carriers, resulting in enhanced release efficacy [255, 258, 259] (Figure 6D). Kang *et al.* used laser 3D bioprinting to produce artificial alveolar barrier matrices consisting of four sequential layers in the following order, endothelial cells, type I collagen, fibroblasts and type I and II alveolar epithelial cells, and the resulting scaffolds were introduced to a TGF-β1 solution, to induce and imitate the phenotype of pulmonary fibrosis [260]. As a next step, both control scaffolds (without the implementation of TGF-β1), as well as the PF biomimicking ones, were treated with nintedanib and pirfenidone, to assess their antifibrotic effect on the bioinks. Sirius red staining after 6 days revealed a very dense collagen network for the PF scaffolds, and a significantly thinner one for the other conditions, especially the pirfenidone one. H&E staining also revealed similar results, with the different layers of the bioink being hardly discernable in the case of the PF scaffolds and pirfenidone again displaying the thinnest layers, indicating its ability to successfully diminish the fibrotic capacity of the PF alveolar barrier structures. Confocal staining for epithelial-mesenchymal transition markers vimentin and nuclear factor-kappa B (NF-kB) p65 were found to depict higher levels of expression in the PF bioinks, compared to control group, as expected. Fibrosis factors fibronectin and α-SMA were investigated, leading to similar results. After the introduction of both drugs into the PF groups, a significant decline in expression of all four markers was evident, with nintedanib having the strongest effect in reducing vimentin and fibronectin formation, while pirfenidone in reducing the levels of NF-kB and α-SMA proteins. RT-qPCR for VIM, MMP9, COL1A1, FN1 and ACTA2 fibrosis-related gene markers were also found to be significantly lower in the presence of both drugs, compared to the PF group.

Bioprinted kidney organoids of differentiated iPSCs on transwell filters (B, top right). Brightfield images on day of printing (Day 7 + 0) to Day 20 of culture (Day 7 + 20), illustrating the spontaneous formation of nephrons over time in micromass cultures. Scale bars are 800 µm (upper panel). Whole-mount immunofluorescence staining of Day 7 + 18 bioprinted organoids, showcasing different cell types within the organoids: podocytes (NPHS1), epithelium (EPCAM), proximal tubular segments (CD13, CUBN, LTL), tubular basement membranes (LAMININ), surrounding stroma (MEIS1/2/3), distal tubule/loop of Henle TAL (SLC12A1), distal connecting segment (GATA3) and endothelium (CD31). Scale bars represent 100 µm (middle panel). Live confocal imaging of a Day 7 + 14 patch organoid derived from the HNF4AYFP reporter iPSC cell line, demonstrating TRITC-albumin substrate uptake (depicted in red) into YFP-positive proximal tubules (depicted in yellow). The images indicate areas of interest within the whole organoid pictures, with and without phase contrast overlays. Scale bars represent 100 µm (lower panel) (reproduced with permission from [238]).

Bioprinting of INS-1 832/3 cells in an alginate/GelMA hydrogel form an extensive vascular network with the support of a PCL mesh and keep their functionality for 9 days *in ovo* and secrete insulin (C, bottom left). CAD model representing the PCL scaffold utilized for 3D printing in the experiments (upper panel, left). A pseudoislet formed from bioprinted INS-1 cells cultivated for 12 days. Immunohistochemical staining of cleaved caspase-3 (brown, asterisk) highlighting apoptotic cells (upper panel, right). *In ovo* chick chorioallantoic membrane (CAM) assay investigated 3D bioprinted INS-1-laden droplets (middle panel). Immunohistochemical staining displaying anti-insulin (brown) and anti-CD31 (red) double-staining of CAM assay explant tissues (lower panel). The rapid vascularization observed in these tissues maintained the viability and function of pseudoislets (reproduced with permission from [246]).

Layer-by-layer 3D bioprinting of lung cancer organoids for drug screening using human pulmonary carcinoma cells (A549) in a HA doped with calcium ions bioink with an alginate/HA/arginine-glycine-aspartic acid (RGD) peptide serving as another ink (D, bottom right). Immunofluorescence analysis of A549 cells on Day 3 depicting the expression of markers pan-cytokeratin (P-CK), prosurfactant protein C (ProSP-C), mucin 1 (MUC1), and caveolin-1 in 2D monolayer and 3D organoid models (reproduced with permission from [259]).

### Co-cultures in bioprinting

Native organs have to meet an excessively complex array of spatial and biological characteristics in order to metabolically prosper. The principal foundation of such a system is based on the establishment of a crosstalk network between the different cell categories that constitute one organ type, which relies on the constant exchange of signals that can control or alter various biomolecular pathways involved in tissue survival and growth [261, 262]. To successfully imitate organ physiology, the parameter of dimensionality has also to be taken into consideration, as cells organically grow unto anisotropic surfaces formed by dense connective tissue of ECM. For this reason, regenerative medicine research shifts away its focus from conventional 2D culture conditions and moves towards the development of 3D culture systems, by implementing more than one cellular type in culture, to better mimic the different organs’ cellular diversity [263–266]. 3D bioprinting is among the most effective biofabrication techniques for the production of artificial whole organ models, as it can facilitate homogenous cell distribution, mimicking that of the real tissue [267], while simultaneously providing the necessary 3D topography for cell proliferation and expression of their biofunctional properties [268].

As mentioned, bone and cartilage are two of the main connective tissue types of musculoskeletal system that display a lot of biomechanical commonalities regarding their supportive role during the movement of the human body and their component’s consistency [269] and even pathophysiology [270] and diagnostic modalities [271]. As such, the development of medical implants designed for the restoration of the osteochondral interface has to cope with great difficulties that stem from the physiological peculiarities of both organs [272, 273]. Osteochondral co-culture 3D bioprinting models have already shown great promise as multicellular regenerative platforms [274, 275]. Qin *et al.* [276] attempted to prepare bilayered dual-function scaffolds, through 3D bioprinting, that could be utilized as substitutes for osteochondral interface reconstitution. Four types of different bioinks were prepared, with the first, acting as control, comprising GG, alginate and methylcellulose (GAM), while the other three, designated as 2LMS-GAM, 5LMS-GAM and 10LMS-GAM, comprised progressively rising concentrations of bioceramic particles bound with Li, Mg and Si ions, aiming to enhance the osteochondral response through their gradual release and subsequent uptake by the entrapped cells. To better imitate the osteochondral interface, the fabricated scaffolds consisted of two layers, with the top one being the result of the mixing of the GAM polysaccharide blend with rabbit chondrocytes (RC) and the bottom one containing a mixture of hMSCs and GAM, with or without the implementation of the bioceramic particles. Over a course of 5 days, all scaffolds showed comparable cell viability, with the integration of the particles having a negligible effect on the bioink rheological properties compared to the control. PCR analysis of various osteochondral markers including aggrecan, SOX9, BMP2, RUNX-2 and COL-1 revealed a significantly higher upregulation in the 5LMS-GAM scaffolds compared to the control ones, validating the particles’ osteochondrogenic impact, a result that was also supported by confocal staining against aggrecan and collagen Type 1, as well as histological analysis of safranin content in the respective scaffolds. Additionally, Bedell *et al.* [29] investigated the effect of multimaterial architecture and the extent of mixing, and thus signaling, locally at the osteochondral interface on the osteochondral integration of 3D bioprinted constructs. More specifically, they examined both gradient and segmented bioprinted fibers encapsulating hMSCs leveraging a bone-like bioink including TCP additives and a cartilage-like bioink including GAG additives and cultured them within a porcine osteochondral explant of 4 mm thickness including a 2-mm cartilage layer and a 2-mm bone layer for three weeks. At the end of the culture period, push-out testing was employed to characterize tissue integration as well as biochemical and histologic analyses to characterize matrix deposition and cell infiltration across the interface. The bone tissue integration for both fiber groups was different from the natural tissue group most probably due to differences in their texture compared to that of trabecular bone. Importantly, both gradient and segmented fiber groups had similar cartilage tissue integration as the natural tissue group, and also the gradient fiber cartilage integration increased by Day 21. These results corroborated with biochemical data of significant sGAG deposition over time in the cartilage section of the gradient fiber group, which was higher per cell than the segmented fiber group.

**Figure 7.**
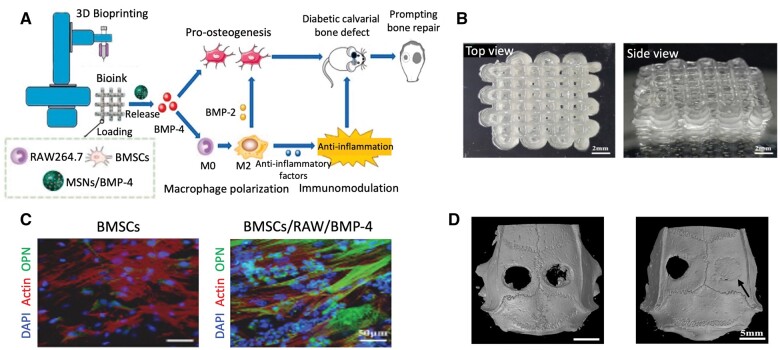
Schematic of bioprinting GelMA/gelatin/PEG and co-culture of bone marrow-derived mesenchymal stem cells (BMSCs) and RAW264.7 macrophages (**A**). Top and side views of 3D bioprinted constructs (scale bars represent 2 mm) (**B**). Osteogenic differentiation of BMSCs within the 3D bioprinted constructs via fluorescence staining for osteopontin (OPN; actin; cell nuclei) after 21 days (**C**, left), and co-culture of BMSCs and RAW264.7 (C, right) (scale bars represent 50 μm). *In vivo* osteogenic assessment of constructs following implantation for 2 months and 3D reconstruction of micro-CT images (black arrow points to the implantation site, scale bars represent 5 mm) (**D**) (reproduced with permission from [279]).

Bone remodeling, either as a result of traumatic fracture, physiological tissue development or various autoinflammatory diseases, is completely reliant on the activity of immunoregulatory system for the correction of balance between bone formation and bone resorption processes. During bone healing, cytokines and other regulatory bioactive species are produced from immune cells and start gathering at the damaged area. Subsequently, the assembly of these molecules can lead to the secretion of bone morphogenetic proteins (BMPs), one of the strongest inducers of mesenchymal stem cell maturation towards osteoblasts [277, 278]. To investigate the crosstalk between BMSCs and RAW264.7 macrophages in a 3D environment, Sun *et al.* [279] prepared bioinks consisting of GelMA, gelatin and PEG, implemented with BMP-4-loaded mesoporous silica nanoparticles (MSNs). The main idea behind this work was that the release of BMP-4 from the nanoparticles, in conjunction with the co-culture of macrophages and mesenchymal stem cells, would induce advanced osteogenic response, while in parallel lead to the suppression of the immunogenic response by guiding the macrophages’ polarization primarily towards the M2 type rather than M1 (Figure 7). Firstly, the team evaluated the role of BMP-4 in macrophages’ polarization in monoculture systems, by preparing two bioinks with RAW264.7 cells, with and without the addition of the BMP-4 loaded MSNs. Fluorescence imaging showed an upregulation of M2 marker (CD206) and suppression of the M1 marker (CCR7) in the case of BMP-4 MSNs containing scaffolds, while PCR analysis also displayed an upregulation of markers related to M2 polarization such as IL-1ra, IL-4 and IL-10 compared to control. The osteogenic capability of the co-culture system was examined by fabricating four types of bioinks of the following consistency, BMSCs, BMSCs/BMP-4, BMSCs/RAW and BMSCs/RAW/BMP-4. Four types of staining were used, two for optical microscopy (alkaline phosphatase activity and Alizarin red staining) and two for fluorescence microscopy (Runx-2 and osteopontin). Out of the four scaffold categories, the BMSCs/RAW/BMP-4 bioinks demonstrated the optimal osteogenic response, validating the authors’ initial hypothesis about the synergistic activity of the two cell populations.

Almost every organ, with some exceptions such cartilage tissue, requires a fully grown vascularized network, whose aim is to act as the main distributor of nutrients that are mandatory for cell survival. Thus, any medical device designed with the intent of being used in clinical medicine ought to facilitate an environment that enables ample vascularization [280, 281]. The growth of organic bone tissue is highly dependent on the development of microvessel formulations. To better mimic the native conditions, during the last years, bone 3D bioprinting has focused more on the production of pre-vascularized bone grafts [18, 282–284]. In an interesting set of work, Byambaa *et al.* [285] created multi-structural GelMA-based 3D printed bioinks, consisting of a perfusable inner soft core of low GelMA concentration, acting as the vasculature vessel, and a more robust outer rim of higher GelMA concentration, to support osteogenesis. Through 1-ethyl-3-(3-dimethylaminopropyl) carbodiimide/*N*-hydroxysuccinimide (EDC/NHS) chemistry, VEGF was also chemically immobilized to GelMA, to further reinforce the angiogenic capacity of the entrapped HUVECs, while silicate nanoplatelets were implemented in the high GelMA concentration outer rim, to induce the osteogenic differentiation of hMSCs towards osteoblasts. To evaluate the inner soft core’s vascularization ability, the team investigated how the length of micro-capillaries structures and their average number of branches formation can be affected by three distinct parameters: (i) low concentration versus high concentration of GelMA, (ii) monoculture of HUVECs against co-culture of HUVECs/hMSCs and (iii) ratio of co-culture cell concentrations, 1:1 and 2:1 (HUVECs: hMSCs). Out of all conditions, over a course of 7 days, the optimal response was demonstrated by the 2:1 low GelMA concentration co-culture bioinks, followed by the 1:1 low GelMA concentration co-culture ones, indicating that both the lower concentration of GelMA, which allows for a faster degradation and thus the formulation of perfusable internal cavities, as well as the co-culture condition of HUVECs and hMSCs can have a significant impact on vasculogenesis. Through alizarin red staining, the role of silicate nanoparticles and the presence of VEGF on hMSCs monoculture was examined at two time points, Day 12 and 21. Although at Day 12, the difference between the various samples was negligible, at Day 21, the bioinks containing both the nanoparticles and the conjugated VEGF had the highest levels of calcium mineralization. Moreover, PCR analysis revealed that most osteogenic markers such as ALP, OCN, OPN and Col1 exhibited significant upregulation levels after the degradation of the low GelMA co-culture inner core, compared to the non-perfusable counterpart, showcasing that the formation of a centrally located hollow lumen containing the two cell populations can drastically alter the osteogenic behavior of the complex bioink system.

Heart tissue activity, as the organ responsible for the ceaseless recycling of blood through the human body, is closely dependent on the establishment of a formed vascularization network. This fact stresses the importance of the development of co-culture systems that could explore and promote cardiac and vascular tissue regeneration in tandem. 3D bioprinting has enabled the facilitation of such holistic models, by adopting techniques that can produce small-scale artificial vascularized ensembles [286]. On that note, Zhang *et al.* [70] taking into consideration the anisotropic structural motifs that govern the native cardiovascular tissue, prepared 3D bioprinted constructs consisting of GelMA, alginate and HUVECs, which were subsequently seeded with rat iPSC-derived cardiomyocytes. The scaffolds were fabricated to architecturally resemble rectangular grids with different aspect ratios of vertical to horizontal lines, leading to four different combinations, 2 × 2, 2 × 3, 2 × 4 and 2 × 5. First, the HUVECs containing bioinks were allowed to incubate for 9 days, in order to proliferate and move towards the opposite poles of the bioprinted fibers and were then seeded with the cardiomyocytes to investigate the spontaneous beating frequencies of the resulting co-culture systems. However, it was found that although the beating of the cardiomyocytes had already begun by the first 3 days of co-culture, presenting similar beating frequencies, independently of the aspect ratio of unit grids, it altogether stopped for the 2 × 2 bioinks by Day 9 and progressively slowed down for the rest of the scaffolds. It is interesting to note that the decline in contractility became less prominent as the levels of anisotropy increased, with the 2 × 5 grids retaining the longest contraction period, validating the group’s initial hypothesis in regards to the contribution that the anisotropic alignment of cardiomyocytes can have on the functionality of cardiac tissue, under a vascularized environment. Additionally, the viability status of the bioinks was explored under perfusion in a bioreactor versus static conditions. As expected, the perfusion condition led to significantly higher biocompatibility, for both cell types.

As mentioned, the liver is a highly complex organ with an anatomical hierarchy that allows for the adoption of multiple biochemical roles, primarily revolving around the constitution and preservation of metabolic homeostasis. Its wide area of biochemical activity relies on its multicellular nature, the development of a fully autonomous vascularized network and the careful orchestration of these two systems. Liver co-culture bioprinting approaches aim to meet those standards, by implementing two or more liver-specific cell populations into 3D structures that would further enable their seamless proliferation and expression of their distinctive biological characteristics [225, 287]. In a most recent study, Cuvellier *et al.* [288] prepared through bioprinting a triple co-culture system including hepatic parenchymal cells (HepaRG), stellate cells (LX-2) and HUVECS, with the aim to design an artificial vascularized liver model. GelMA was selected as the base material for the bioink formulation which was photopolymerized in the presence of LAP. Bioprinted constructs containing only HepaRG cells were cultivated up to 28 days, to assess their fibrotic directionality as opposed to 2D HepaRG control monocultures supplemented with dimethyl sulfoxide (DMSO). Confocal staining microscopy for E-Cadherin, N-Cadherin and MRP2 fibrotic markers validated the gradual development of circular fibrotic like lobules, peaking at Day 28, but at a lesser degree compared to control, probably due to the limited available space that the engulfed cells have for expansion inside the bioinks. PCR analysis for the hepatogenic markers ALB, ALDOB, HNF4A, NR1H4, NR1/2 and SERPINA1 also depicted comparable values to those of control. The LX-2 myofibroblastic expression was correlated to the effect of the presence of TGFβ-1 in monoculture conditions. Myofibrosis-related genes such as ACTA2, COL1A1, TIMP1 and MMP2 showed significant upregulation compared to scaffolds not having been implemented with TGFβ-1, as well as compared to 2D control, especially at Days 9 and 16. Finally, for the evaluation of the triple co-culture, collagen production of bioinks containing HUVECs, HepaRG and LX-2 cells was compared to that of HepaRG monoculture, LX-2 monoculture and HepaRG plus HUVECs co-culture scaffolds, once again with and without the presence of TGFβ-1. Out of all respective combinations, triple co-culture enriched with TGFβ-1 had the highest expression of COL1A1 gene. Surprisingly, collagen staining revealed that the triple co-culture bioinks without TGFβ-1 had a more pronounced collagen network formation than those with TGFβ-1. Table 3 presents various works revolving around 3D co-culture bioprinting, along with their contribution significance.

**Table 3.** A summary of the development of various works around co-culture bioprinting, based on cell type used, combination of biomaterials, bioprinting technique and scientific relevance

Tissues	Cell types	Biomaterials	Technique(s)	Significance	Ref
Bone, cartilage	hMSCs	GelMA + XG + NFC + β-TCP or HAMA	Extrusion/inkjet/DLP	Bioink components affect the expression of bone and or cartilage markers of hMSCs.	[29]
Bone + cartilage	hpMSCs + RCs	GG + Alg + MC + LMS	Extrusion	Different cells can be simultaneously prompted towards specific tissue due to the presence of bioceramics.	[276]
Bone	BMSCs + RAW	GelMA + Gel + PEG + MSNs	Extrusion	BMP-4 release can polarize RAW towards M2 macrophages and enhance the osteogenic response of BMSCs.	[279]
Bone + endothelium	hMSCs + HUVECs	GelMA	Extrusion	The existence of a central angiogenic vessel enhances the formation and maturation of bone tissue and medium perfusion upregulates osteogenic genes.	[285]
Endothelium + cardiac	HUVECs + rat cardiomyocytes	GelMA + Alg	Extrusion	Engineered cardiac organoids can be fabricated by adjusting the macroscopic anisotropy of the bioprinted microfibrous construct, resembling the bundled structure of the myocardium *in vivo*.	[70]
Liver + endothelium	HepaRG + LX-2 + HUVECs	GelMA	Extrusion	HepaRG cells differentiate in a DMSO-free medium while stellate cells remain at their ‘uiescent’ stage	[288]

hMSCs, human mesenchymal stem cells; GelMA, gelatin methacryloyl; XG, xanthan gum; NFC, nanofibrillated cellulose; β-TCP, beta-tricalcium phosphate; Hama, methacrylated hyaluronic acid; hpMSCs, human placental mesenchymal stem cells; RCs, rabbit chondrocytes; GG, gellan gum; alg, sodium alginate; MC, methylcellulose; LMS, Li-Mg-Si bioceramics; RAW, RAW264.7 macrophages; gel, gelatin; PEG, polyethylene glycol; MSNs, mesoporous silica nanoparticles; HUVECs, human umbilical vein endothelial cells; HepaRG, hepatic parenchymal cells; LX-2, stellate cells.

### Current challenges and future outlook

The development of artificial single-organ systems, as well as their *in vitro* and *in vivo* evaluation, presents a tremendously challenging task to undertake due to the intricate biomechanical nature of organic tissue. Although 3D bioprinting has emerged as one of the most auspicious technologies for the refinement of the production process of tissue engineering platforms, it has also been met with skepticism in regards to its true advantageous nature over other biofabrication techniques.

Currently, 3D organ bioprinting is faced with a multitude of regulatory obstacles that overshadow its applicability, among them being, the long-term unpredictable nature of the bioprinted constructs, limited animal pre-clinical testing data, social inequality that can stem from overpriced services and lack of concrete legal framework in regards to 3D bioprinting as a medical implant producing methodology and its corresponding intellectual properties. As most bioinks are quite sensitive and suffer from stability issues, one possible side effect after the fabrication of the bioprinted construct is its premature failure due to mechanical damage or their non-sufficient immobilization to the site of interest. In the latter case, translocation to impertinent regions of the human body can occur, rendering their therapeutic potential fully redundant. Additionally, pre-clinical testing in animals or other humans evokes both bioethical and health-safety issues, since results from medical trials on different specimens than those that they are targeted for, cannot guarantee a personalized medicine technique’s efficacy with high precision. As 3D bioprinting is a relatively expensive medicinal approach, its utility in medical practice is bound to allow for limited accessibility for the economically lower classes, with even the possibility for the emergence of cases of 3D organ trafficking in the near future. To successfully alleviate most of these problems, organizations such as Food and Drug Administration (FDA) and European Medicines Agency (EMA) are obligated to provide a full legislation plan, in an attempt to incorporate very strict guidelines as to how 3D organ bioprinting should be conducted in everyday medical practice, define the prerequisites that need to be met in order to be considered as a completely safe methodology for human transplantation, as well as to investigate different avenues as to how the accessibility chasm between the different social strata could be resolved, before its full-scale commercialization [289].

In order for a bioink to be considered for clinical use, it ought to allow for ample vascularization and innervation, two conditions that are pivotal to secure its successful integration into the adjacent tissue target. The lack of a fully autonomously acting vascular and neural network cannot support the long-term metabolic needs of a biofabricated construct, limiting its overall regenerative capacity [290, 291]. Another important risk parameter is the provoking of immunogenic reaction by the bioinks components, whether that stems from the materials or the cells themselves, leading to the ineluctable rejection of the transplant [292].

To address these hurdles, as mentioned in the co-culture bioprinting subsection in greater detail, recent 3D bioprinting approaches should focus more on employing novel methodologies that can facilitate the necessary microenvironment for the biofabrication of whole organ models [286, 293]. One of the currently governing issues in regards to whole organ bioprinting is the rather limited upscalability, as well as the difficulty of production of bioinks that can act as uniform platforms for the simultaneous expansion of multicellular cell lines. For this reason, bioinks consisting of multiple layers have been designed, to better accommodate the needs of each cell type, while also enabling intercellular communication [134, 285]. However, the monitoring of the biological response of such multi-level co-culture structures can be quite taxing, especially the *in vivo* evaluation. Additionally, 3D bioprinting has showed great prospects as an alternative avenue for the delivery of immunogenicity-related molecules [46, 294] and as tumor replicating platforms for drug screening applications [295–297]. Of particular interest is the bioprinting of immunomodulatory constructs encapsulating macrophages, as these cell types have shown to not only act as one of the chief regulators for immunogenic response, but also directly control chemotaxis-related processes of cell differentiation and tissue regrowth [279, 298, 299].

Hydrogel stability and gradual degradation over prolonged periods of time are essential for any successful medical device. Since bioink components have to be water soluble, the arsenal of available biomaterials for their construction is severely limited. This fact stresses the need for the investigation of new biomaterial combinations. Additionally, even if a bioprinted construct exhibits desirable biological attributes, it might still suffer from poor mechanical properties, resulting in restricted applicability [51]. However, harder materials not only lead to the jeopardization of the printing process by evoking immiscibility issues with the other constituents or premature physical gelation during the extrusion phase but can also significantly reduce cell viability due to the strangulating effect of higher extrusion pressures [32, 300]. One of the principal methods of mechanical reinforcement and stabilization of hydrogel scaffolds is physical and chemical crosslinking, which can leave chemical traces behind that can depict cytotoxic behavior [301]. For the preparation of bioinks, this effect can be adverse towards the initial cell population viability, as 3D bioprinted constructs can be washed only a finite number of times in order to remove toxic residuals, without damaging the engulfed cells. As such, novel bioink stabilization approaches are required to be further investigated that could allow their utilization as feasible medical implants.

One of the more promising bioprinting methods that aim to combine optimal biological response while simultaneously retaining good printing fidelity is suspended bioprinting [302, 303], in which the printing process is conducted upon a sacrificial substrate bath, that can be easily removed afterwards, usually through mild heating, leaving the 3D bioprinted scaffold completely intact. This method enables the facile printing of soft hydrogels that exhibit tunable mechanical properties, without sacrificing printing resolution, giving ample space for biomechanical adaptation [303, 304]. Lan *et al.* [305] managed to 3D bioprint scaffolds for nasal cartilage regeneration through suspended bioprinting by employing collagen type I, a material with excellent biological properties but notoriously limited printing capacity. Visible light photopolymerization is a favorable type of chemical crosslinking which has gathered much interest lately as a tool for scaffold stabilization in 3D bioprinting, as it allows for the printing of soft bioinks at low extrusion pressures, with or without some degree of pre-crosslinking, thus causing less cytotoxicity, while also giving the opportunity for further post-printing irradiation of the structures to achieve desirable mechanical strength, with miniscule impact on cytocompatibility. As opposed to older, more conventional UV-light-based photopolymerizable systems, it employs photoinitiators such as eosin Y [306], Ru/SPS [56], riboflavin [307] and graphitic carbon nitride [308], which are activated at visible light wavelength spectrum (above 380 nm), ensuring less detrimental effects on cells and thus higher biocompatibility levels.

Although additional progress is required to make 3D bioprinting, a viable option as an established method of personalized medicine therapy from a technical point of view, one of the biggest concerns of the scientific community regarding its applicability range stems from the overcomplicated nature of the technique itself, the inconvenient production process and the overall high cost of its components. Stem cells and growth factors are two of the most prominent constituents for 3D bioprinting. Most stem cell lines are usually very expensive to obtain and maintain, while usually presenting difficulties during the harvesting procedure. Some of these issues include anatomical particularities that do not allow for easy entry to the site of interest, a limited number of available cells and possible contaminations of the samples. Another significant downside of 3D bioprinting is the lack of time-responsiveness, referring to the current inability of the technique to cope with the on-demand and mass production of bioinks designed for specific therapeutical purposes. This is due to the fact that it is extremely challenging to successfully synchronize the timeline that is necessary for the expansion of multiple cell lines in large quantities with the immediate need for a patient to be surgically operated, as imposed by their respective health condition. Additionally, the operation of all bioprinting-related devices, encompassing both the different varieties of 3D bioprinters and bioreactors, requires trained medical professionals, with extensive expertise in the field.

To overcome technological barriers before achieving the capacity to produce fully autonomously functional organs, 3D bioprinting needs to focus on the development of techniques that enable upscaled production of 3D bioprinted constructs that maintain their structural hierarchy. They should also establish novel cell expansion systems to cope with the need for significant cell numbers required for the preparation of a bioink designed for *in vivo* applications. One of the most cutting-edge technological achievements in 3D bioprinting is the development of multi-modal bioprinting, which constitutes the synchronous 3D bioprinting of different bioink materials, at the same time, striving for augmented biomimicry levels. This multi-material bioprinting approach allows the conjunction of multiple 3D bioprinting modalities such as multi-nozzle extrusion, inkjet and different methods of vat-photopolymerization bioprinting, which can in turn amplify production complexity, in terms of both design and biological properties [309, 310]. The emergence of machine-learning has also been recently looked favorably as a coping tool for various adversities that 3D bioprinting is currently faced with. Usually, identification of the ideal printing parameters that would ensure both good printability and low cell mortality can be very lackluster, requiring multiple trial and error experiments which cost both money and time. Data-driven machine learning algorithms can impactfully hasten the investigation process for the ideal bioink traits, by delving into vast data collections, accruing particular information based on the desirable properties, and finally conferring an adept predictive model, even before commencing any further *in vitro* experiments [311, 312].

Moreover, protocols that would allow the long-term storage of bioinks should substantially contribute to their further utilization and commercialization as medical implants. One such emerging methodology designated as cryobioprinting, involves the mixing of cryopreservant agents with bioprinting solutions, making them viable for on-demand use, without compromising their biological response [313]. Capitalizing on that novel approach, Ravanbakhsh *et al.* [314] used cryobioprinting to examine the printability, cell viability and differentiation capacity of GelMA-based scaffolds. The research group focused on the investigation of how the aforementioned properties are affected when DMSO and melezitose are used as cryoprotectants in tandem. It was revealed that when a combination of 10% w/v DMSO and 12% w/v melezitose concentrations was used, after the thawing of the scaffolds, cells exhibited great levels of resuscitation, while retaining their differentiation potential regardless of cell type. Even more interestingly, cryobioprinting proved to amplify printing fidelity, compared to conventional 3D bioprinting. All these findings strongly support the method’s validity as an innovative technique for the preparation of shelf-ready implants.

Before 3D bioprinting is integrated into medical systems as a viable method of personalized medicine treatment, a stronger link between medical professionals and scientific specialists must be established. The complexity of the technique, due to its intricate technical nature at the frontiers of interdisciplinarity, necessitates fluent cooperation and open communication among medicinal institutions and healthcare providers with research and technology facilities, universities and biotechnology companies. Although contemporary health systems have started to shift their philosophy towards more holistic models, by recruiting and involving varying and multidisciplinary teams in their arsenal, we are still far away before 3D bioprinting could fully manifest as a potential therapeutic approach in patients. Table 4 presents an overview of the current challenges and suggested solutions for organ bioprinting.

**Table 4.** A Brief overview of the main challenges that contemporary bioprinting technologies need to treat before they can find broader utilization in clinical practice

Contemporary 3D organ bioprinting challenges		Solutions
Lack of concrete legal framework for 3D organ bioprinting elicits regulatory issues.	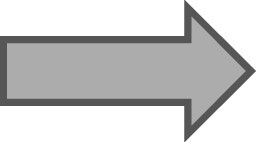	Organizations such as FDA and EMA should provide strict legislation plans revolving around 3D bioprinting safety standards and its applications in everyday medical practice.
Monoculture examination is not sufficient to evaluate complex mechanisms of native tissues.	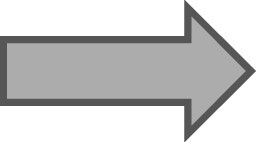	Development of co-culture bioprinting systems to better resemble native tissues.
Soft bioinks are preferable to maximize cell viability during bioprinting, however, they suffer from low printing resolution and limited stability.	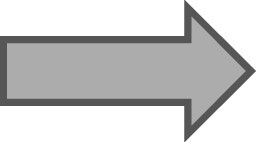	New techniques such as FRESH or visible light-based photopolymerizable systems can negate the brittleness of soft bioinks, without jeopardizing cell viability and printing accuracy.
3D bioprinting requires significant optimization times due to technological barriers and a finite number of available biomaterials.	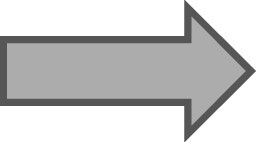	Machine-learning approaches can impactfully hasten the investigation process for the ideal bioink components, based on the particularities of the tissue type we aim to restore.
Limited upscaling capability and involvement of complex methodologies requiring expensive equipment and consumables.	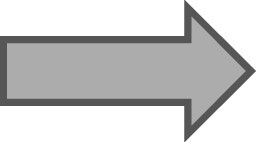	Novel technologies allow for long term storage of bioinks such as cryobioprinting and the establishment of stronger communication between healthcare and scientific personnel.

## Conclusions

This review highlights the recent advances in 3D organ bioprinting, one of the fastest-growing fields in tissue engineering, due to its unique ability to produce structures of versatile architecture, tailorable mechanical strength and biological affinity. We primarily focused on describing the biomaterial properties that define the prerequisites for regeneration-competent bioinks, and, based on these criteria, presented recent research endeavors on the utilization of various 3D bioprinting techniques to produce cell-laden constructs with biomimicking properties targeting several different organ types. Taking into account that native tissues are complex and consist of diverse cell populations and distinct structural layering, we discussed the role of co-culture in bioprinting, which constitutes one of the next steps in the evolution of organ bioprinting, further amplifying its feasibility. Moreover, we expanded on the current limitations of organ bioprinting, while providing insightful suggestions on how they could be overcome before it could be considered for personalized medicine applications.
